# Effect of genomic distance on coexpression of coregulated genes in *E*. *coli*

**DOI:** 10.1371/journal.pone.0174887

**Published:** 2017-04-18

**Authors:** Lucia Pannier, Enrique Merino, Kathleen Marchal, Julio Collado-Vides

**Affiliations:** 1 Programa de Genómica Computacional, Centro de Ciencias Genómicas, Universidad Nacional Autónoma de México, Cuernavaca, Morelos, México; 2 Departamento de Microbiología Molecular, Instituto de Biotecnología, Universidad Nacional Autónoma de México, Cuernavaca, Morelos, México; 3 Department of Microbial and Molecular Systems, KU Leuven, Centre of Microbial and Plant Genetics, Leuven, Belgium; 4 Department of Plant Biotechnology and Bioinformatics, Ghent University, Technologiepark, Ghent, Belgium; 5 Department of Information Technology, Ghent University, IMinds, Ghent, Belgium; 6 Department of Genetics, University of Pretoria, Hatfield Campus, Pretoria, South Africa; Hosei University, JAPAN

## Abstract

In prokaryotes, genomic distance is a feature that in addition to coregulation affects coexpression. Several observations, such as genomic clustering of highly coexpressed small regulons, support the idea that coexpression behavior of coregulated genes is affected by the distance between the coregulated genes. However, the specific contribution of distance in addition to coregulation in determining the degree of coexpression has not yet been studied systematically. In this work, we exploit the rich information in RegulonDB to study how the genomic distance between coregulated genes affects their degree of coexpression, measured by pairwise similarity of expression profiles obtained under a large number of conditions. We observed that, in general, coregulated genes display higher degrees of coexpression as they are more closely located on the genome. This contribution of genomic distance in determining the degree of coexpression was relatively small compared to the degree of coexpression that was determined by the tightness of the coregulation (degree of overlap of regulatory programs) but was shown to be evolutionary constrained. In addition, the distance effect was sufficient to guarantee coexpression of coregulated genes that are located at very short distances, irrespective of their tightness of coregulation. This is partly but definitely not always because the close distance is also the cause of the coregulation. In cases where it is not, we hypothesize that the effect of the distance on coexpression could be caused by the fact that coregulated genes closely located to each other are also relatively more equidistantly located from their common TF and therefore subject to more similar levels of TF molecules. The absolute genomic distance of the coregulated genes to their common TF-coding gene tends to be less important in determining the degree of coexpression. Our results pinpoint the importance of taking into account the combined effect of distance and coregulation when studying prokaryotic coexpression and transcriptional regulation.

## Introduction

Transcriptional coregulation in general implies coexpression: genes that are regulated by the same Transcription Factors (TFs) are more likely to be coexpressed. RegulonDB defines the transcriptional programs of genes in *E*. *coli* K-12 based on curated information. A distinction is often made between simple and complex transcriptional regulatory programs depending on whether a gene’s regulatory program consists of at most one or more TFs. Genes are defined to be **coregulated** if their respective regulatory program overlaps, i.e. if they are coregulated by at least one TF with the same role (activator, repressor or dual). The complexity of their individual regulatory programs in combination with the extent to which their program overlaps defines **the tightness of the coregulation**. Genes with a completely identical regulatory program are expected to be more tightly coregulated under all conditions than in case of an incomplete overlap. In the latter case different gene-specific TFs can be involved in tuning the expression at the individual gene level (less tight coregulation). Also if more TFs are shared by the coregulated genes, their coregulation can be expected to be tighter.

Evidence exists that besides coregulation also the genomic distance between two genes contributes to their coexpression. Closely located genes are more coexpressed than faraway located genes in *E*. *coli* [1,2], yeast [3,4], *Arabidopsis* [5], zebrafish [6] and humans [5]. Several mechanisms supporting coexpression behavior of closeby located genes have been reported in prokaryotes, including operonic organization, bidirectional cotranscription at divergent promoters [2,7,8] and genomic clustering of highly coexpressed small regulons, i.e. of TFs such as GntR and GadW that only regulate a few operons [9–11]. These observations suggest that coregulation and genomic vicinity both can contribute to the degree to which two genes tend to be coexpressed. However, assessing the contribution of the genomic distance added to coregulation in determining coexpression is complicated as in many cases the close distance between genes is also at the basis of their mechanism of coregulation (genes located in the same operon, read-through transcription of contiguous operons [12], and bidirectional cotranscription at divergent promoters [2,7]). In this study we exploited the large body of information in RegulonDB together with publicly available expression data to systematically assess whether the genomic distance affects the degree of coexpression, independently of the coregulation mechanism.

We tested to what extent the distance between coregulated genes is associated with their degree of coexpression. Our results confirm that genomic vicinity of coregulated genes is an important factor that contributes to higher levels of coexpression, also for genes that are not tightly coregulated. This observation was further supported by the finding that there was an evolutionary constraint in maintaining the distance between coregulated genes that are highly coexpressed.

## Results

### Assessing the degree of coexpression between coregulated genes

In bacteria, genomic distance between genes is a feature that, in addition to coregulation, affects coexpression. In this study, we aimed at assessing whether and how genomic distance between coregulated genes associates with their degree of coexpression. The degree of coexpression between genes was assessed by calculating the pairwise similarity between their gene expression profiles obtained from a large scale expression compendium assessing expression under 4077 condition contrasts (Materials and methods) [13].

To identify the measure that best reflects the degree of pairwise coexpression between any pair of coregulated genes we tested six similarity measures based on respectively correlation and mutual information (see Materials and methods and Supplementary file S1 File part 1). The measure referred to as Spearman Correlation Rank (SCR) performed best in separating the coexpression behavior of genes that were expected (genes within the same operon) to be highly coexpressed from those that were not (genes not known to be coregulated). In addition, we could show that this rank-based measure better normalized for the unequal number of samples present in the compendium that represent the conditions under which the different TFs are active, as explained in detail in the Supplementary File S1 File part 2.

In the remainder of the analysis the degree of coexpression between two genes is thus defined as the pairwise similarity between the expression profiles of these genes as measured by SCR. High SCR values between two genes correspond to a low degree of coexpression whereas low SCR values correspond to a high degree of coexpression.

To gain a first insight into the overall degree to which coregulated genes are coexpressed, we calculated their average degree of coexpression using SCR (Supplementary File S1 File part 3). In the context of this study, coregulated genes were defined as any set of two genes that have at least one common TF in their respective regulatory programs with the same regulatory effect on each of the considered genes (activation, repression or both). Whether two genes were coregulated was derived from curated information on TF-gene regulatory interactions in RegulonDB [14] (Materials and methods). We deliberately excluded pairs of coregulated genes originating from the same operon as for operonic transcription, coregulation and distance are confounded (i.e. the closeby location is the cause of the coregulation) and including these operonic coregulated genes would blur assessing the effect of the genomic distance between coregulated genes on their degree of coexpression.

We observed that on average, the degree of coexpression between genes known to be coregulated was rather low, as was also previously reported [15]. In particular, genes coregulated by a common *global* TF (here defined as a TF with more than 130 target genes), but not by any other additional common more specific TF, showed a relatively low degree of coexpression. Those coregulated genes that only have a global TF in the common part of their regulatory program were excluded from further analysis as they are known to be only loosely coregulated (Materials and methods) and including them results in underestimating the average degree of coexpression between coregulated genes. In Supplementary Table S1 Table we provided a full list of 91 TFs that together control 11339 pairs of coregulated genes considered in this study, as well as per TF the mean pairwise genomic distances and the mean degree of pairwise coexpression between the target genes coregulated by that TF.

### Distance between coregulated genes inversely correlates with the mean degree of coexpression

We hypothesized that the distance between coregulated genes has an influence on their coexpression degree. To test this hypothesis, we examined the relationship between the pairwise genomic distance between coregulated genes and their degree of coexpression. The pairwise linear distance between genes along the circular chromosome, hereafter referred to as *distance*, was determined by the number of base pairs separating the start positions of two genes.

**Fig 1:**
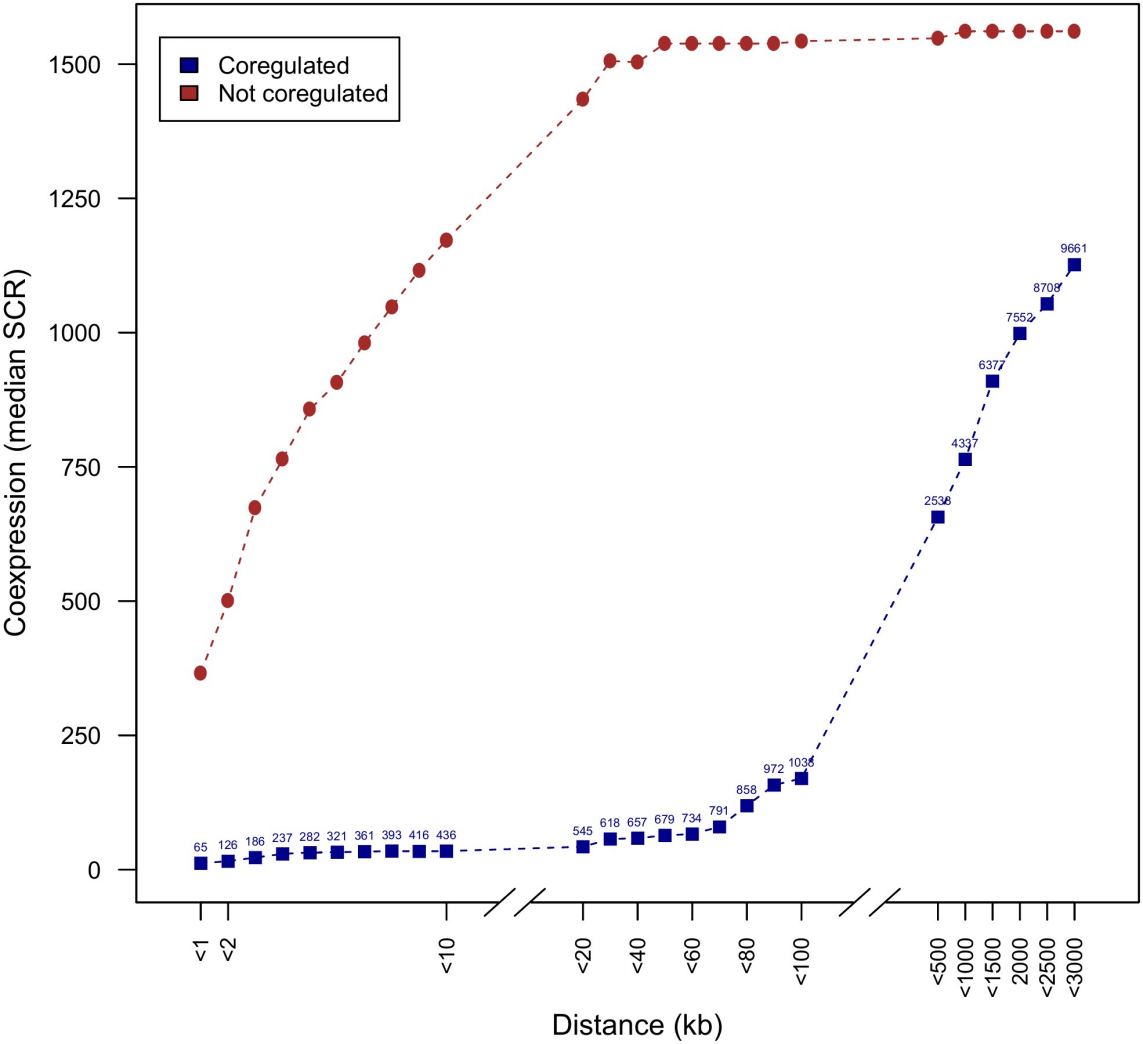
The distance between coregulated genes negatively influences their coexpression degree. The plot shows the mean coexpression degree as a function of the maximum distance between two genes. The distance (x-axis) is measured by the number of kb (kilo base pairs, equal to 1000 base pairs) between the structural gene start positions of two genes. Coexpression degree (y-axis) is measured by the median SCR (a low median SCR implies high degree of coexpression) of genes with a distance lower or equal to the distance indicated on the x-axis. The effect of distance on coexpression is shown for all coregulated genes (dark-blue curve). Coexpression degree of coregulated genes can be compared to the negative control containing all genes not known to be coregulated (red curve). Note that breaks in the x-axis between distances <10 and <20 kb and between distances <100 and <500 correspond to scale differences. The numbers above each data point of the dark-blue curve represent the number of pairs of coregulated genes for which the median SCR was calculated.

In Fig 1 the mean degree of coexpression is shown as a function of the distance between genes, i.e., the median SCR (y-axis) of a pair of genes for which the distance between the two genes is smaller than a given value (x-axis). The mean coexpression degrees between genes that were not known as coregulated was shown as a negative control (Fig 1, red curve).

Overall, we observed a clear influence of the distance on the degree of coexpression: coregulated genes tend to be pairwisely more coexpressed when they are closely located than when they are more distantly located (see Fig 1, slope of dark-blue curve). Also in the negative control (genes not known to be coregulated) at small distances (see slope of red curve, distances <20 kb) a relative high degree of coexpression was observed. Because genomic clustering and coexpression tend to be associated [16], genomically colocalized genes might tend to be more coexpressed, irrespective of whether they are coregulated by the same TF. According to Sobetzko et al. [16], colocalization of genes tends to trigger some degree of coexpression because at close distances, levels of DNA supercoiling tend to be similar, hereby leading to similar gene expression patterns. So genes that are clustered on the genome might therefore be coexpressed as a mere result of their closeby positioning rather than because of coregulation. To test whether this was indeed the case, we have identified genes that belong to distinct regulons (i.e. genes regulated by distinct TFs) that are genomically colocalized with each other (Materials and methods). We have compared the degree of coexpression of pairs of colocalized genes that were also coregulated versus the degree of coexpression of gene pairs that were colocalized but not coregulated. We still observed a significant difference in degree of coexpression between both gene classes (Kruskal-Wallis p-value << 0.001), indicating that genomic colocalization alone most likely cannot be responsible for the high degrees of coexpression observed for some gene pairs in the (negative) reference set.

It thus is more likely that the relatively high degree of coexpression in the negative control at small genomic distances is the result of the incompleteness of the information in RegulonDB rather than being the consequence of the small distance: because of missing information in RegulonDB, we cannot exclude that a minor fraction of these so-called non-coregulated gene pairs are in fact coregulated. Further analysis (data not shown) indeed showed that the observed relatively high average coexpression degree of non-coregulated genes at small distances visible in Fig 1 could be attributed to a small fraction of the non-coregulated genes showing high degrees of coexpression but that the majority of the non-coregulated genes were not highly coexpressed. An additional overlay of the set of genes reported to be non-coregulated and having high degrees of coexpression with sets of genes that were predicted to be in vitro coregulated based on SELEX results [17] confirmed that indeed several of the so-called non-coregulated genes with high degree of coexpression might actually be coregulated (listed in Supplementary Table S2 Table).

### The effect of the distance on coexpression decreases as the tightness of the coregulation increases

**Fig 2:**
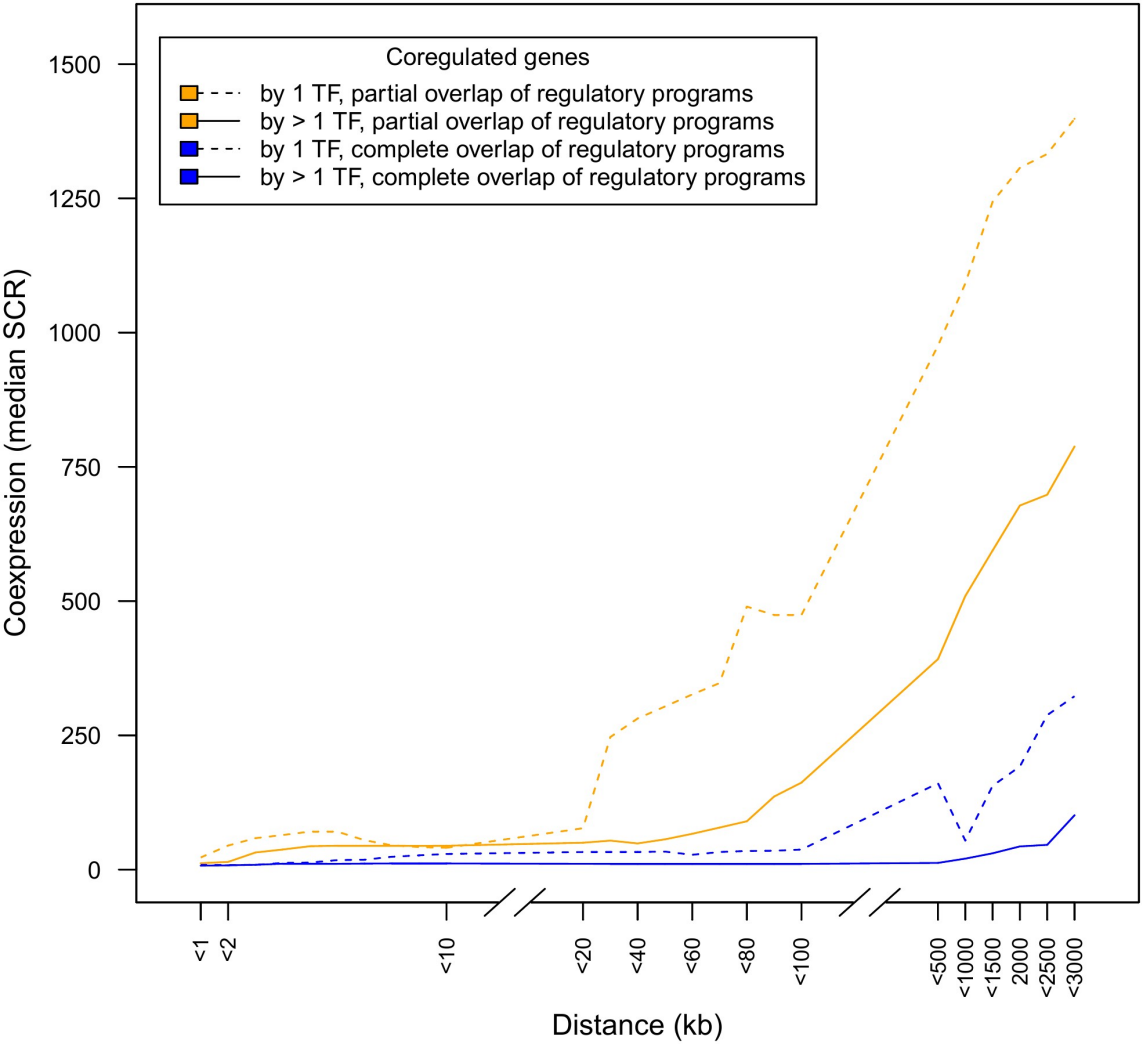
The distance between coregulated genes has a larger influence on their coexpression degree if genes are less tightly coregulated. The coexpression behavior of coregulated genes was disentangled, depending on whether the regulatory programs displayed complete versus partial overlap (blue versus orange) and depending on the number of common TFs present in the overlapping part of their regulatory program (dotted line for 1 TF versus full line for >1 TF).

To assess whether the effect of the distance in determining the degree of coexpression was dependent on the coregulation tightness, we first subdivided coregulated genes in two groups depending on whether **their regulatory programs overlapped completely versus partially**: if two coregulated genes have a completely overlapping regulatory program they are assumed to be more tightly coregulated than when their regulatory programs are only partially overlapping. A partial overlap means that at least one of the coregulated genes has TFs in its regulatory program that are not shared by the other gene or when the same TF has different effects on each gene. Indeed, as shown in Fig 2 the degree of coexpression between coregulated genes with complete overlap of regulatory programs is higher than that of coregulated genes with only a partial overlap of regulatory programs and that this is true over all distances considered (blue versus orange curve). Regarding the effect of distance on the degree of coexpression, this effect exists for both genes that have completely overlapping versus those that have only a partially overlapping regulatory program. However the distance effect is most pronounced for genes that have a partially overlapping program but lasts at larger distances for genes with a completely overlapping regulatory program (respectively around <20 kb versus around <100 kb).

In addition, we made a distinction between genes **that are coregulated by one versus those that are coregulated by more than one TF**, as we assume that coregulation by multiple common TFs can also contribute to a larger coregulation tightness with a possible effect on the degree of coexpression [18]. The effect of the coregulation tightness determined by the number of common TFs in the overlapping part of the regulatory programs is confounded with the degree to which the regulatory program overlaps (e.g. it is hard to compare the degree of tightness of coexpression between a partial overlapping program with three shared TFs and a completely overlapping program with one TF). Therefore we conditioned the effects of the number of TFs in the shared part of their regulatory programs on whether the regulatory programs of these coregulated genes were completely versus partially overlapping. Both in case of a complete or a partial overlap of regulatory programs, we observed a higher degree of coexpression for those genes that have more than one common TF than for those that have only one common TF in the overlapping part of their regulatory programs. Also both in case of complete and partial overlap of regulatory programs, the degree of coexpression remains higher at larger distances for genes with more than one common TF versus for those with just one common TF (Fig 2, full orange curve versus dotted orange curve for partial overlap of regulatory programs and full blue curve versus dotted blue curve for complete overlap of regulatory programs).

In general, distance thus affects the degree of coexpression, irrespective of the tightness of coregulation. For the most tightly coregulated genes the effect of distance is less visible as the genes tend to be highly coexpressed anyway and thus the contribution of small distances in increasing the degree of coexpression is the least pronounced for the most tightly coregulated genes. This indicates that the effect of distance is relatively small compared to effect of the tightness of the coregulation in determining the degree to which coregulated genes are coexpressed.

### Non-operonic adjacent genes that are coregulated show a high degree of coexpression independently of their coregulation tightness or their genomic orientation

Focusing on coregulated genes that are located in each other’s close neighborhood (<1 kb to <20 kb), it seems that their degree of coexpression is almost independent of the tightness of their coregulation: at such small distances, the mean degree of coexpression is not significantly different for coregulated genes with a completely overlapping or a partially overlapping regulatory program, and not significantly different for coregulated genes that share one or that share more TFs in the overlapping part of their regulatory program (Kruskal-Wallis p << 0.001, see also Fig 2, for respectively orange versus blue, and full versus dotted lines).

We argued that for genes that are involved in the same biological processes but are the *least tightly coregulated* i.e. by 1 TF and not the same regulatory program, their nearby location might be a way to guarantee the high degree of coexpression that would be needed to make them available together. To assess whether this was true in our data, we assessed whether indeed the least tightly coregulated genes that are located nearby were associated more frequently to the same biological processes than the least tightly coregulated genes located at larger genomic distances (>10 kb); to associate genes to biological processes Gene Ontology (GO) annotations were used (Materials and methods). This seemed indeed to be the case (Kruskal-Wallis p = 0.007).

From Fig 2 also appears that at an *extremely small* distance between coregulated genes (<2 kb), a high degree of coexpression of the coregulated genes is almost guaranteed irrespective of the tightness of coregulation (except for the very least tightly coregulated genes, see orange dotted curve). However, at such small distances we cannot exclude that the observed high degree of coexpression is caused by the occurrence of shared promoter elements (in divergently oriented adjacent promoters), or, *read-through* transcription [12] or not yet annotated operons (in codirectionally oriented promoters).

As these alternative causes of the observed high degree of coexpression can only exist for cases of divergently and codirectionally oriented gene pairs, we tested to which extent the high degree of coexpression observed between coregulated genes located at small distances from each other also held for convergently oriented genes.

Hereto we analyzed how the coexpression of genes that are members of coregulated adjacent operons, referred to as *coregulated proximally located genes*, depends on their relative orientation. Proximally located genes with divergent orientation are overrepresented in our dataset compared to those with other orientations (368 out of 490 proximally located pairs of genes or 75%) supporting the idea that divergent orientation indeed has evolved as a prevalent mechanism of assuring coexpression between adjacent coregulated genes as was also described by Korbel et al. [2]. Our results reveal that indeed proximally located coregulated genes are highly coexpressed when divergently oriented (median SCR 47). Also codirectionally oriented proximally located coregulated genes are highly coexpressed as expected (median SCR 44). Having a divergent or codirectional promoter orientation can thus definitely account for part of the observed high degree of coexpression between proximally located coregulated genes. However, interestingly, also proximally located coregulated genes with convergent orientation showed equally high coexpression as those with the divergent and codirectional orientation (median SCR 34): coexpression was not significantly different between the divergent, codirectional or convergent orientation as indicated by the Kruskal-Wallis test (p = 0.84).

This observation indicates that at proximal distances, not only with distance confounded mechanisms of coregulation such as bidirectional cotranscription, readthrough transcription or unannotated operons, but also mere close distance can account for the observed high degrees of coexpression, independently from coregulation tightness. Note that the latter conclusion relies heavily on the evidence of 30 pairs only of proximally located genes in convergent orientation. One might argue therefore that we cannot rule out that the high degree of coexpression observed for coregulated genes at small distances is not the mere consequence of confounded mechanisms such as readthrough transcription.

To specifically assess the effect of readthrough transcription we evaluated whether the degree of coexpression of coregulated genes that are not proximally located but still located at small distances, was significantly lower than that of proximally located genes (with 'small distance' being defined as an intergenic distance of maximally 12 kb, equal to the maximum distance that is observed between proximally located genes). The mean degree of coexpression of coregulated genes that are not proximally located (145 pairs of genes) is not significantly different from that of proximally located genes (490 pairs of genes) (Kruskall-Wallis p = 0.41), indicating that besides known mechanisms, such as read-through transcription, also the mere effect of the small distance plays a role in determining levels of coexpression.

So, given that the relative orientation does not bias the coexpression degree of proximally located genes we conclude that the relative orientation causes no bias for the observed effect of the distance on the degree of coexpression of coregulated genes.

### Coregulated genes are more coexpressed when they are located equidistantly relative to their common TF coding gene

To find a potential mechanism by which close distance of coregulated genes that is not mediated by read-through transcription or bidirectional cotranscription can explain higher degrees of coexpression, the following reasoning was made: assuming that the availability of TF molecules is limited by diffusion and assuming that coregulated genes that are exposed to similar quantities of TF proteins will be more coexpressed than coregulated genes that are not, we reasoned that coregulated genes that are more equidistantly located from their common TF coding gene are exposed to a more similar quantity of the TF encoded gene product and as a consequence will tend to be more coexpressed than coregulated genes that are not located equidistantly from their common TF gene.

To test this assumption, we compared the degree of coexpression hereby distinguishing between 1) coregulated genes located equidistantly with respect to their common TF gene and 2) coregulated genes not located equidistantly to their common TF gene. Equidistant means that the two distances, i.e. between the common TF gene and the two target genes, are within 90% of one another (Materials and methods). We restricted the analysis to genes that are coregulated by at most one TF in order to unequivocally define equidistancy to one and the same common TF and to exclude possible interferences of distances to other common TFs.

**Fig 3:**
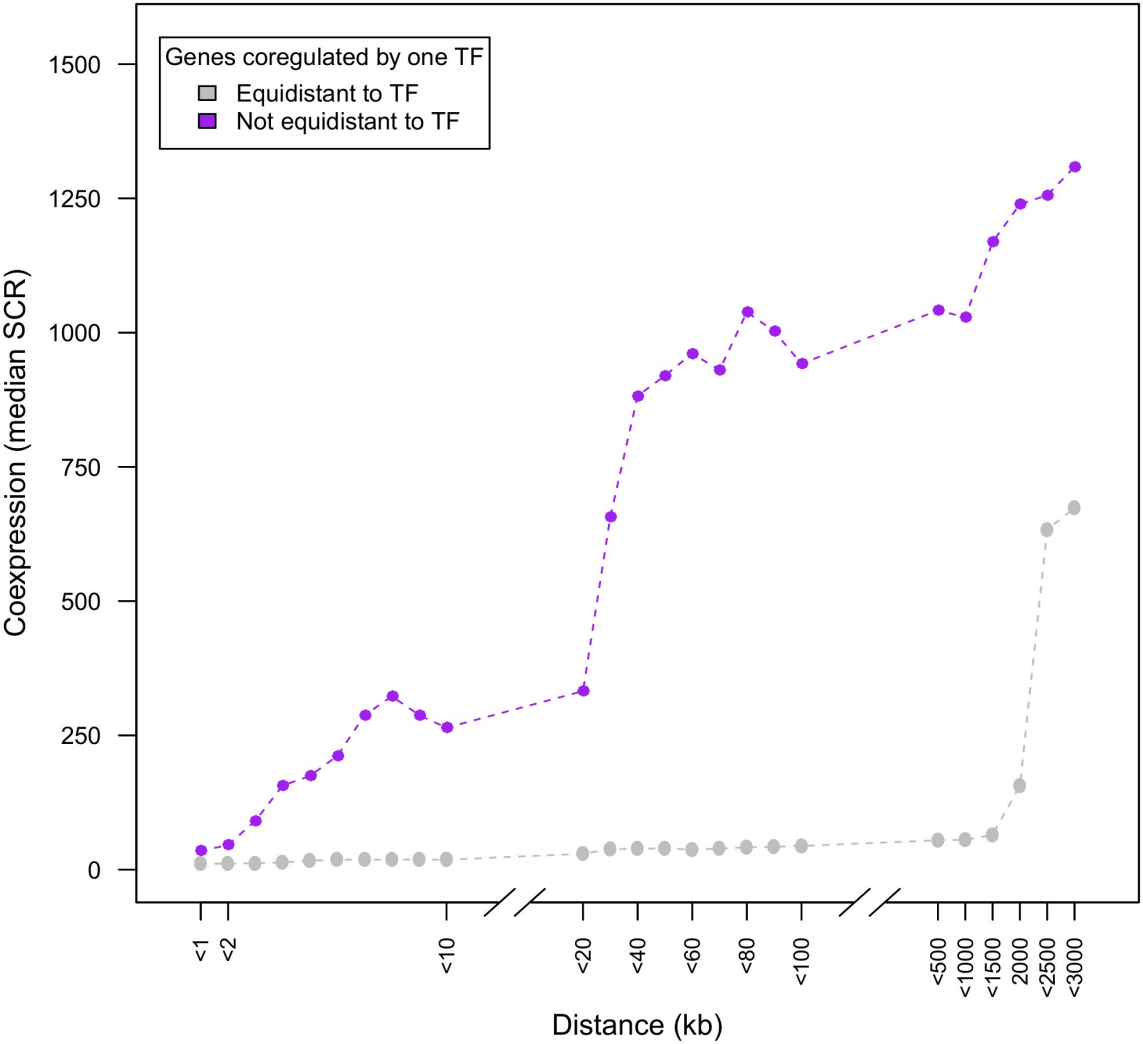
Effect of relative distance between TF and target genes on the degree of coexpression of the target genes. The coexpression behavior of genes that are coregulated by one TF is disentangled, depending on whether genes are equidistantly located (grey) or not equidistantly located (purple) relative to their common TF-coding gene. Y-axis displays the degree of coexpression (SCR), X-axis displays the maximum genomic distance between the coregulated genes.

Fig 3 shows that coregulated genes that are equidistantly located from their common TF(s) (grey curve) generally are more coexpressed than genes that are not equidistantly located (purple curve).

### The degree of coexpression between coregulated genes does not depend on the nearby location of their common TF coding gene

The previous paragraph supported the hypothesis that coregulated genes located equidistantly from their common TF are subject to similar local quantities of TF proteins and therefore show a higher degree of coexpression. One could also hypothesize that the closeness of the TF coding gene itself could result in higher absolute local TF quantities in the target neighborhood, and as such increases the degree of coexpression of coregulated genes.

Fig 3 however shows that coexpression remains remarkably high for coregulated genes that are located equidistantly from their common TF, even when the genes themselves are located relatively distant from each other and thus by definition also relatively further from their common TF. This implies that for tightly coregulated genes sharing one common TF, coexpression is not only independent of the distance between those genes but, as a consequence, also independent of the distance of those genes relative to their common TF coding gene.

We further statistically tested this independence of the degree of coexpression on the distance between the common TF coding gene and the coregulated target genes. Hereto coregulated genes were classified in two groups referred to as *near to TF* or *far from TF*, depending on whether the distance between the common TF and the coregulated targets was smaller than or larger than 30 kb, respectively (Materials and methods). We included in these groups only those coregulated genes that were (1) located equidistantly from their common TF in order to study the mere effect of the distance between the TF and the coregulated genes on the degree of coexpression and to exclude the effect of unequal distances between the common TF and coregulated targets (see previous paragraph) and (2) coregulated by at most one TF to exclude effects caused by multiple common TFs between the coregulated genes or the effect of additional TFs that were not shared by the analyzed coregulated genes. Interestingly no statistically significant difference in degree of coexpression was observed between the two groups of coregulated genes referred to as respectively *near to TF* or *far from TF*, i.e. the null hypothesis of the Kruskal-Wallis test was rejected and the SCR of *near to TF* and *far from TF* are samples that come from the same population (p = 0.50).

In conclusion, our results demonstrate that in contrast to equidistancy from a common TF, a closer distance of a common TF to coregulated genes does not result in a higher degree of coexpression.

### Close distance between highly coexpressed coregulated genes is evolutionarily constrained

Here we assumed that if the distance between coregulated genes plays a key role in affecting the degree of coexpression between those coregulated genes, this distance should be evolutionarily constrained. To test this assumption, we performed a comparative study in the subclass of gamma-proteobacteria [19–21] to assess whether the distance between coregulated genes is evolutionarily more conserved when the coregulated genes display a high degree of coexpression than when they do not. We started the analysis using all pairs of coregulated genes in *E*. *coli* and determined the orthologous of those genes in other gamma-proteobacteria. We defined as metric of *distance conservation* the proportion of the number of ortholog gene pairs in the different species for which genes have a distance equal to or smaller than the distance between the two corresponding coregulated genes in *E*. *coli* on the total number of considered orthologous pairs (Materials and methods).

**Fig 4:**
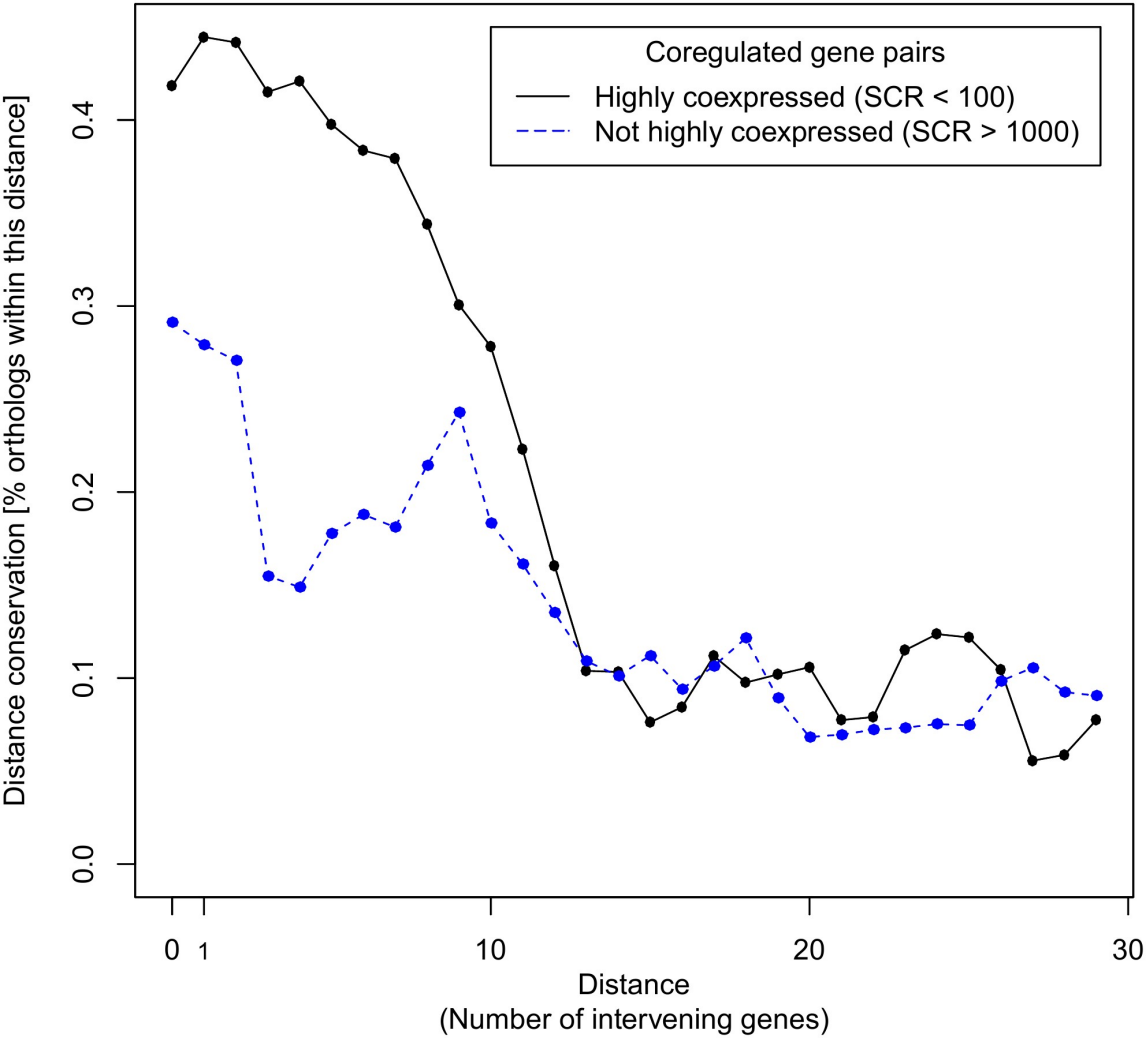
Evolutionary conservation of distance between coregulated genes. The x-axis represents the pairwise genomic distance between coregulated genes in E. coli, measured in intervening genes. The y-axis represents the degree to which for coregulated genes in E. coli the genomic distance is evolutionarily conserved in other gamma-proteobacteria which is expressed as the fraction of orthologous gene pairs for which the distance is equal or smaller (y-axis) than the distance between the corresponding genes in E. coli (x-axis) over the total number of analyzed orthologous genes. Orthologous genes are pairs of genes in other species that are orthologous to a pair considered in E. coli, i.e. a pair of coregulated genes in E. coli is expected to have an orthologous counterpart in other gamma-proteobacterial species if both genes in the E. coli pair have an orthologous counterpart in the considered gamma-proteobacterial species. Results are shown for respectively pairs of genes that are highly coexpressed (SCR < 100) (black curve) versus pairs of genes that are not coexpressed (SCR > 1000) (blue curve).

In Fig 4 we plotted the average distance conservation of highly coexpressed (SCR < 100) and not (highly) coexpressed (SCR > 1000) coregulated genes in *E*. *coli* as a function of the distance between the genes. It can be observed that coregulated genes located at small distances (< 10 intervening genes) have a stronger distance conservation when they are highly coexpressed (30–40%, black curve) than when they are not coexpressed (25–30%, blue curve). This observation indicates that for highly coexpressed genes located in each other’s neighborhood on the genome there is an evolutionary constraint on conserving their small distance. Because evolutionary conservation of close distance of genes has been associated with horizontal gene co-transfer [22], we further hypothesized that highly coexpressed genes that are nearby located and that show strong distance conservation are likely to show evidence of horizontal co-transfer. We indeed found evidence of horizontal gene co-transfer for several of these cases (see Supplementary File S1 File part 5) which is thus an additional indication that for highly coexpressed nearby located genes there exists an evolutionary constraint for maintaining their small distance.

These results provide further evidence that for nearby coregulated genes for which coexpression is crucial, the vicinity or small distance is a driving force for guaranteeing high coexpression.

## Discussion

In prokaryotes, genomic distance is a feature that in addition to coregulation affects coexpression. In this work, we evaluated how the genomic distance of genes known to be coregulated in *E*. *coli* contributes to their coexpression behavior. Hereto, we combined information on regulation in *E*. *coli* K-12 reported in RegulonDB, one of the largest curated and continually updated transcriptional databases, with publicly available expression data. Based on the information available for *E*. *coli* K-12 we observed that in general coregulated genes display higher degrees of coexpression as they are more closely located on the genome.

For genes that display very tight coregulation (e.g. genes with the exact same regulatory programs), this additional effect of genomic vicinity on coexpression is less pronounced compared to the distance effect observed for genes that are less tightly coregulated. This indicates that the contribution of genomic distance in determining the degree of coexpression is relatively small compared to the degree of coexpression that was determined by the tightness of the coregulation. As a consequence especially for non-tightly coregulated genes, distance seems to have a critical role in guaranteeing coexpression: only when located at small distances, the effect of the common TFs in increasing coexpression is large enough to compensate for the effect of the non-common TFs in potentially lowering coexpression. We found indications that non-tightly coregulated genes are located nearby to guarantee high coexpression in order to coordinate their common involvement in a particular biological process.

We showed that at very small distances, coexpression is high irrespective of the tightness of coregulation. This is because the small distance is at least partially the cause of coregulation, as is the case for *read-through* transcription or potentially unannotated operons (in codirectionally oriented operons) or for bidirectional cotranscription through common regulatory elements (in divergently oriented operons). However genes located in convergently oriented operons are also found to be highly coexpressed. In the latter case, the small distance cannot be causal to the coregulation and thus supports the idea of a *distance effect* as an additional factor independent of coregulation triggering high coexpression of closeby located coregulated genes.

We hypothesized that part of the distance effect can be explained by the fact that coregulated genes that are more closely located to each other are subject to more similar levels of TF molecules and are therefore more highly coexpressed. We could support this hypothesis by showing that coregulated genes that were located at similar distances relative to their common TF tend to be more coexpressed than genes that were not located equidistantly relative to their common TF. At very small distances, coregulated genes were found to be highly coexpressed, irrespective of whether or not they are located equidistantly relative to their TF. This may be explained by the fact that both coregulated genes are so close to each other that their distance to the common TF can only slightly differ.

Unlike the distance between target genes, the distance of the targets to the common TF coding gene does not seem to play a major role in determining coexpression of coregulated genes. This shows that, even when limited TF diffusion [23] may reduce TF availability at distances far away from the TF coding gene, coexpression can still be guaranteed because both targets are subject to a minimal, but comparable quantity of TF proteins. This hypothesis assumes that both target genes have the same response to their common TF, i.e. an equal concentration of TF proteins is needed to trigger gene expression (in the case of an activator TF) or to inhibit gene expression (in the case of a repressor TF). Even though the assumption of equal responses to a TF in different target genes seems a major simplification of reality, for example because of different affinities or different numbers of binding sites for the common TF, in general, the effect of distance on coexpression is still visible.

Alternatively, one could imagine that when promoter regions reside at small distances, they are likely to be subject to the same degree of DNA supercoiling, bending or looping and thus more equally accessible to common TFs than more distantly located promoter regions [16,24–29]. In addition, colocalized promoter regions are more likely to be subject to the same degree of RNA polymerase molecules and the same degree of DNA phosphorylation which may add to the tightness of coregulation of nearby genes and thus to their coexpression. The observation that nearby coregulated genes tend to conserve their close distance more if they are highly coexpressed further adds to the importance of the vicinity in driving coexpression.

It is important to remark that our definition of *distance* being the linear distance along the chromosome is a strong simplification of the dynamic three-dimensional (3D) genome structure. As we currently do not have sufficient data available on dynamic 3D distances between genes, it is difficult to know the effect of the 3D distances. However, given that TF diffusion not only happens through 3D space but also by one-dimensional movement of TFs along the DNA segment such as “sliding” and “hopping” [30], it is not surprising that we find that also simply the linear genomic distance is a critical factor for coexpression of coregulated genes.

In conclusion, we systematically demonstrated that as much as genes are controlled by common TFs, their genomic distance functions is an additional and independent factor determining their coexpression. Our assumption that TF accessibility seems to be an important cause for enhancing coexpression at small distances, opens the door to more studies on local levels of TF molecules and their role in driving coexpression. In future studies on transcriptional regulation, distance is a critical factor to be taken into account in driving coexpression.

## Materials and methods

### Expression data

To retrieve *E*. *coli* expression data, we used the publicly available large-scale expression compendium COLOMBOS v3.0 compiling 4077 condition *contrasts* for 4321 genes [13]. ‘Condition contrasts’ do not represent single experimental conditions, but represent the difference between a test and reference condition (the differential expression values between the respective test and reference conditions in a particular contrast is expressed as a logratio). This concept ‘condition contrast’ is used in COLOMBOS to render expression values comparable across platforms and experiments. A full list of growth conditions from which the contrasts were derived as well as a more detailed explanation for condition *contrasts* is available at www.colombos.net.

### Operon definitions

Operons were taken from direct literature curation at RegulonDB and bioinformatics predictions from ProOpDB [31]. Operon architectures were taken from ProOpDB because the accuracy of predictions of this database is one of the highest reported thus far (94.6%). In addition, the operon prediction of this database did not include coexpression as information source, whereby we avoided any circularity problem.

### Coexpression measure

To quantify the degree of coexpression between any two genes, all pairwise similarities between gene expression profiles across all experimental conditions of the expression compendium were calculated. We tested six different similarity measures: Pearson Correlation Coefficient (PCC), Spearman Correlation Coefficient (SCC), Mutual Information (MI), Pearson Correlation Rank (PCR), Spearman Correlation Rank (SCR) and Mutual Information Rank (MIR) and selected SCR as the similarity measure for our study as explained in Supplementary File S1 File part 1.

Note that our assessment of coexpression only took into account positive correlation and no anticorrelation. Although theoretically an inverse correlation could be expected, for example, between a repressor TF and its target genes, based on this work and our previous experience [10] it appears that negative correlation coefficients are not at all common. We therefore deliberately omitted assessing negative correlations as they would contribute relatively more spurious associations than true correlations.

The PCR, SCR and MIR, mentioned above are rank-based derivatives of respectively the PCC, SCC and MI and quantify how similar the expression profiles of two genes are relative to how similar these genes' expression profiles are to the expression profiles of all other genes (i.e. the similarity of expression profiles measured by PCC, SCC and MI respectively). The calculation of these rank-based derivatives of the PCC, SCC and MI is based on the work of Obayashi and coworkers [32,33]. In their work they propose the ‘mutual rank’ which is the ranked derivative of the PCC (here referred to as PCR). Below we provide details on the derivation of the SCR from the SCC according to the procedure described by Obayashi et al. [33]. The derivation of the PCR from the PCC and the MIR from the MI is calculated analogously. The derivation of the SCR from the SCC is as follows: calculating the SCC results in a symmetrical matrix in which each value contains the Spearman correlation between the gene expression profiles of any two genes A and B. (Supplementary File S1 File, part 2). This SCC matrix is converted into an asymmetric ranked matrix. To this end we assign a rank to each value in the row direction of the correlation matrix i.e. we rank all correlation values of gene A where the lowest rank i.e. 1 is assigned to the highest SCC value of gene A in the row and further ranks are assigned in descending order of the row SCC values of gene A. Each ranked value thus expresses how correlated gene A is with gene B relative to its correlation with all other genes (see Supplementary file S1 File part 2). This results in an asymmetric matrix in which the rank assigned to the correlation of gene A to B is not necessarily the same as the rank assigned to the correlation of gene B to A.

For each gene pair A-B an *SCR value* is subsequently derived by calculating the geometric mean of the two ranked values of A-B and B-A. We used the geometric mean rather than the arithmetic mean as this performed better as a measure of coexpression; this has been proved by Obayashi et al [33] and also showed the best results on our benchmark (data not shown). The added value of these rank-based derivatives of correlation in assessing the degree of coexpression between genes was described in the work of Obayashi and colleagues [33] and the advantage of using these measures particularly in our setup is explained in the Supplementary File S1 File part 2.

### Modes of coregulation

Sets of coregulated genes were derived from regulatory interactions derived in RegulonDB v9.0 [14], a database containing information on the transcriptional regulation of *E*. *coli* strain K-12. Depending on the information that is available to support TF-gene regulatory interactions, RegulonDB distinguishes between interactions with “strong” or “weak” evidence. To ensure that the results derived in the main text were not influenced by whether or not we included interactions with weak evidence, we tested the impact of using different sets of interactions on our results, more specifically we tested a set including all interactions (i.e. those supported by weak plus those by strong evidence i.e. a total of 3430 interactions), a set excluding interactions supported by one type of weak evidence only (2961 interactions), and a set containing interactions based on strong evidence only (in comparison to the previous setting here also interactions that are supported by two types of weak evidence are excluded, i.e. 2213 interactions). Results of these tests are presented in (Supplementary Material part 4) and show that in general the results and general conclusions hold irrespective of the type of dataset that was used as input. In the main text the results are shown for a dataset that containing all interactions except those supported by at most one source of weak evidence as this dataset offers a trade-off between containing the most reliable interactions, but still being sufficiently large to make statistical inferences.

Starting from the defined 2961 interactions, we derived 76891 coregulated genes used for our analysis; these were selected by taking all combinations of two genes that are not in the same operon (known and predicted operons as described above) and share at least one common TF with the same regulatory effect (activation, repression or dual). In total, 56235 out of 76891 coregulated gene pairs were coregulated only by a global TF and were left out: TFs with at least 130 target genes were considered global TFs, i.e. CRP (380 target genes), FNR (150), IHF (131), ArcA (133), Fis (268), and H-NS (140). The filtered dataset contained 11399 pairs of genes that are coregulated by at least one of 91 non-global TFs. A full list of the 91 TFs along with the number of pairs of genes they coregulate and per TF the mean of all pairwise distances and mean degrees of coexpression between the genes coregulated by that TF is given in the Supplementary Table S1 Table. Genes with a complete overlap of regulatory programs are defined as pairs of coregulated genes for which all TFs known to be involved in the regulation of either gene and with the same role (activator, repressor, or dual) are shared between both genes. Genes with a partially overlapping regulatory program are pairs of coregulated genes that do not share all of the TFs known to be involved in their regulation.

### Distance measures

The distance between two genes is equal to the shortest distance (in base pairs) between the two structural gene start positions, i.e. by taking the shortest distance along the circular chromosome. Hereby, the shortest distance between two genes by definition is always smaller than half the chromosome length (4600 base pairs or 4,6 kilo base pairs). Note that for the assessment of distance conservation a different measure of distance was used (see below).

### Measure of average degree of coexpression

In the plots of Figs 1–3 we took the median SCR as a measure of average coexpression degree because the median is less susceptible to *outliers* (here pairs of genes with extreme low degrees of coexpression (which means a high value of SCR) than the mean.

### Identification of equidistancy to TF coding gene

To analyze the effect of equidistancy and the effect of distance to the common TF on coexpression of coregulated genes, we only considered genes that are coregulated by one common TF (1238 pairs of genes) to exclude additional and/or confounded effects due to coregulation by multiple TFs. Coregulated genes were considered to be located equidistantly from their common *TF* (i.e., TF coding gene), if the proportion of the smallest and the largest of the two corresponding distances for each of the two genes to the TF exceeded 0.9. Pairs of genes for which this was not the case were considered to be not equidistantly located relative to their common *TF*. In total 122 pairs of genes were located equidistantly and 1116 pairs of genes were located not equidistantly to their common TF coding gene.

When both genes in a pair have a distance to the common *TF* coding gene that was < = 30 kb the gene pair was considered to be located near to their common TF coding gene or *near to TF*. Alternatively if both genes in the pair had a distance to the common TF coding gene that was >30 kb the pair was considered to be located far from the TF coding gene or *far from TF*. A cut-off of 30 kb was taken by plotting the median SCR as a function of the distance of both target genes to the TF coding gene; at a distance of 30 kb, the slope of the median SCR changes, i.e., the rate at which the degree of coexpression decreases with the distance becomes lower (data not shown), 30 kb thus determines the range below which the effect of the distance on coregulation is most visible.

### Measure to assess functional similarity

To assess whether pairs of genes belonged to the same functionality class according to Gene Ontology (GO) we used the BioConductor package GOSemSim [34] that allowed calculating the degree to which similar GO terms were associated to the considered pairs of genes. Gene Ontology annotations were downloaded from the gene ontology website (http://geneontology.org/page/go-annotation-file-format-20) and GO similarity between genes was calculated by taking semantic similarity between GO terms within the “Biological Process” ontology that were associated to the genes.

### Identification of colocalized regulons

To identify different sets of coregulated genes that were genomicallycolocalized, we selected a set of coregulated genes between which the distance genes was <10 kb. Coregulated gene pairs within this set were used to calculate the degree of coexpression of colocalized coregulated genes. To assess the degree of coexpression of colocalized non-coregulated genes we used the combinations of genes from the set that were colocalized but did not share the same TF. The Kruskal-Wallis test was used to assess differences in mean degree of coexpression between the two sets.

### Evolutionary conservation of distance

All genes and distances (as measured by the number of intervening genes) of 267 species of gamma-proteobacteria were collected with their respective orthologs for each gene in *E*. *coli* from GeConT [35]. In GeConT, two genes were considered to be orthologs by using Bidirectional Best Hit [36]. For each pair of coregulated genes with distance D in *E*. *coli*, we extracted N orthologous pairs of genes (with distance d) in N of 286 gamma-proteobacterial species, i.e., species in which orthologs existed for both genes of the *E*. *coli* pair. Conservation of the distance or *distance conservation* was defined as the proportion of orthologous pairs with distance d< = D relative to the total number of orthologous pairs, with orthologous pairs being the pairs of genes in a given species which are orthologous to two coregulated genes in *E*. *coli*. To select orthologous pairs we only considered species for which both genes in a coregulated pair of genes in *E*. *coli* contained an orthologous counterpart. For the evaluation of this metric, we considered the *distance* between any two genes as the *number of intervening genes* to normalize for the fact that the length of intergenic regions between orthologous genes can differ in different organisms. For the selection of highly and not highly coexpressed coregulated genes we took pairs of coregulated genes with SCR < 100 (5347 pairs of genes) and with SCR > 1000 (54936 pairs of genes), respectively.

## Supporting information

S1 File This file contains the following sections:
- Selection of a similarity measure to quantify coexpression- Rank-based similarity measures compensate for conditional dependency- The degree of coexpression of genes that are coregulated in E. coli is generally low- Evidence of horizontal gene co-transfer in genes with strong distance conservation
(DOC)

S1 Table List of TFs that control the pairs of coregulated genes used in this study. This table shows all TFs considered in our analysis with at least one pair of coregulated genes (see Materials and methods for the definition of coregulated genes). For each TF we showed the number of pairs of genes coregulated by that TF, the mean distance between every two genes in a pair (in base pairs), and the mean coexpression (as measured by SCR).
(DOC)

S2 Table List of so-called non-coregulated genes that are potentially coregulated as derived from SELEX. This table shows an excerpt of pairs of genes that belong to the negative control of genes not known to be coregulated but highly coexpressed and located nearby and that were predicted to be coregulated according to SELEX. Gene 1 and gene 2 correspond to a pair of genes selected from the set of so-called non-coregulated genes with selection criteria 1) small distance (< 10 kb) 2) high degree of coexpression (SCR<10) and 3) at least one common TF in their respective set of TFs as predicted by SELEX. The coexpression degree between gene 1 and gene 2 is given in SCR. One or more predicted common TF(s) was (were) given. The numbers between brackets refer to the Nth best hit (which means Nth highest % similarity of that TF for that gene) that TF was for respectively gene 1 and gene 2.
(DOCX)

S3 Table List of all coregulated genes with their genomic distances and coexpression degrees. This table contains the following fields: TF (coregulating TF), gene 1 (first gene in the pair), gene 2 (second gene in the pair), distance (genomic distance between gene 1 and gene 2), TF-gene1_distance (genomic distance between the TF and the first gene), TF-gene2_distance (genomic distance between the TF and the second gene), role (a = activator, r = repressor, d = dual), SCR (Spearman Correlation Rank as a measure of coexpression degree).
(TXT)

## Abbreviations

AUC: Area Under the Curve
MI: Mutual Information
MIR: Mutual Information Rank
PCC: Pearson Correlation Coefficient
PCR: Pearson Correlation Rank
SCC: Spearman Correlation Coefficient
SCR: Spearman Correlation Rank
TF: Transcription Factor
TFBS: Transcription Factor Binding Site
TN: True Negative
TP: True Positive
