# Supplementary material for: Effect of genomic distance on coexpression of coregulated genes in E. coli
Source: PLoS One. 2017 Apr 18;12(4):e0174887. doi: 10.1371/journal.pone.0174887 (PMC5395161; doi:10.1371/journal.pone.0174887)
Supplement: S1 File — This file contains the following sections: - Selection of a similarity measure to quantify coexpression- Rank-based similarity measures compensate for conditional dependency- The degree of coexpression of genes that are coregulated in E. coli is generally low- Evidence of horizontal gene co-transfer in genes with strong distance conservation (DOC) [file pone.0174887.s001.doc]

**1. Selection of a similarity measure to quantify coexpression**

To select the similarity measure that best captured the degree of coexpression between two coregulated genes in our setting, we compared six similarity measures, i.e. three similarity measures commonly used to quantify coexpression, the Pearson Correlation Coefficient (PCC), Spearman Correlation Coefficient (SCC) and Mutual Information (MI), and their rank-based derivatives, which we defined as respectively the Pearson Correlation Rank (PCR), Spearman Correlation Rank (SCR) and Mutual Information Rank (MIR) (the calculation of these measures was explained in Materials and Methods).

As a benchmark, we used genes located within the same operon (using all combinations of genes within the same operon according to the operon set of RegulonDB [1]), as these are expected to be highly coexpressed. To exclude the effect of regulatory elements within operons, we only considered in the benchmark pairs of operonic genes that are contiguous and that are not separated from each other by an internal promoter or terminator.

This resulted in a positive set of 602 gene pairs (N = 602) which were expected to be highly coexpressed. These were referred to as constituting the True Positive (TP) set. As a negative control, 10000 random gene pairs were sampled and referred to as True Negative (TN) set. For both the positive and negative set, we calculated the PCC, SCC, MI, PCR, SCR and MIR across all conditions in COLOMBOS.

As an illustration, Fig 1 shows the frequency distributions, i.e. the number of gene pairs, of respectively the TP and TN sets that were coexpressed within a given range of the SCC and within a given range of SCR (its rank-based derivative). SCR values of contiguous operonic genes are localized at the utter left of the SCR distribution which is the most significant region, while the SCR distribution of the TN set (genes in random pairs) is uniform.

***Fig 1. Comparison of the SCC and SCR in assessing the degree of coexpression.*** *When using the SCR as a measure of the degree of coexpression between pairs of genes belonging to the same operon, the assessed degree of coexpression is consistently high. SCC (A) and SRC (B) distributions based on expression data from the COLOMBOS compendium for a set of random gene pairs (TN) (yellow) and contiguous within operon gene pairs (TP) (blue). Each bar plot shows two histograms representing the coexpression distribution for TP gene pairs (N = 1,238) (explained in the text) and TN random gene pairs (N = 1,000).*

In contrast, for the SCC the majority of TP pairs have a degree of coexpression that ranges from approximately 0.1 to approximately 0.7. In contrast to what is observed for the SCR, for the SCC the majority of TP seem to cover a large range of values: 86% of TP gene pairs have an SCC within the interval [0.25, 0.75], which covers 50% of the full positive range of SCC (positive range is the range that looks at correlation and not at anticorrelation i.e. [0-1]), whereas 86% of TP gene pairs have an SCR within the interval [1, 30], which covers only 7% of the full range of SCR.

This means that according to the SCR the majority of TP gene pairs are highly coexpressed, whereas when assessing the coexpression with the SCC it is intuitively more difficult to interpret whether the true positives have a relatively high or low coexpression degree.

This was formally confirmed by assessing the performance of each measure for its ability to classify TP (within operonic genes) and TN (random gene pairs) based on their degree of coexpression. This ability was quantified by calculating the Area Under the Curve (AUC) from the ROC curve (Table). The AUC equals the probability that a classifier will rank a randomly chosen positive instance higher than a randomly chosen negative one. In other words, the higher the AUC is, the better the measure is able to separate TP pairs from TN pairs based on their differences in coexpression behaviour.

In Table 1 the Area Under the Curve (AUC) is given for each of the six tested similarity measures as a quantification of how well each measure distinguishes between the TP and TN gene pairs. The highest AUC (0.998) corresponded to SCR using the corresponding distributions of the TP and TN pairs. This implies that TP and TN can be best separated using their coexpression behaviour measured by SCR.

|  | PCC | SCC | MI | **PCR** | **SCR** | **MIR** |
| --- | --- | --- | --- | --- | --- | --- |
| AUC | 0.9738 | 0.9888 | 0.9609 | **0.9816** | **0.9933** | **0.9726** |

***Table 1.*** ***Area Under the Curve (AUC) as a performance measure for the similarity measures PCC, SCC, MI, PCR, SCR, and MIR.*** *The AUC calculated from the ROC curve quantifies the ability of a measure to separate TP from TN, in this case a measure of coexpression to separate contiguous pairs of operonic genes from random gene pairs. The first three columns of Table 1 represent PCC, SCC and MI, and the next three columns represent their corresponding PCR, SCR and MIR values.*

Overall, because SCR a) performs best in distinguishing TP from TN (Table 1), b) provides a measure of coexpression behaviour that is more comparable and interpretable between gene pairs (Fig 1) SCR was used as coexpression measure in all our analyses.

**2. Rank-based similarity measures compensate for conditional dependency**

The rank derivatives of the standard used PCC, SCC and MI inherently normalize for the variability in ranges of PCC, SCC and MI values that can be observed between genes in a given dataset and hereby facilitate comparing degrees of coexpression between gene pairs.

Consequently part of the reason why the SCR, as a rank-based derivative of the more classically used Spearman Correlation Coefficient performed so well in our study is it’s improved ability to compensate for the conditional dependency of transcriptional regulation than the standard used coexpression measures, such as PCC, SCC or MI. In our study, coexpression between genes was measured across all experiments of the expression compendium, irrespective of the conditions under which the genes were effectively coexpressed and thus assumed to be coregulated. When using standard correlation measures such as PCC, SCC or MI, genes that are coregulated under a low number of conditions only because of sample biases in the compendium, will by definition exhibit a low degree of measured coexpression [38]. As a result with standard coexpression measures, such as PCC, SCC or MI it is difficult to distinguish between a low degree of coexpression and/or coregulation or a high degree of coexpression and/or coregulation that was observed in a small subset of the conditions only. Both situations give rise to low measured degrees of coexpression. For the rank-based derivatives of the PCC, SCC or MI on the contrary this is less of an issue, as they express the expression similarity of one gene versus the other gene in a gene pair (i.e., A versus B) relative to the expression similarity of both A versus all other genes and B versus all other genes as mentioned. Thus, even when two genes are highly coexpressed in a small subset of the conditions only, their SCR value might still be equally high as that of genes that are coexpressed under a large set of conditions. Therefore, rank-based derivatives of PCC, SCC or MI are expected to be more robust against biases in the number of samples of specific conditions in the compendium.

Our results show that this is indeed the case (Fig S2): in our positive control, pairs of genes that are supposed to be coexpressed consistently receive consistently high coexpression values (low SCR) when using a coexpression measure based on the SCR whereas the range of their Spearman correlation values (SCC) is much wider. For instance for the two operonic genes essD and rrrD, the SCC is 0.28 (low correlation which means a low degree of coexpression degree) whereas the SCR of 3.87 (i.e. a low SCR which means high coexpression degree).


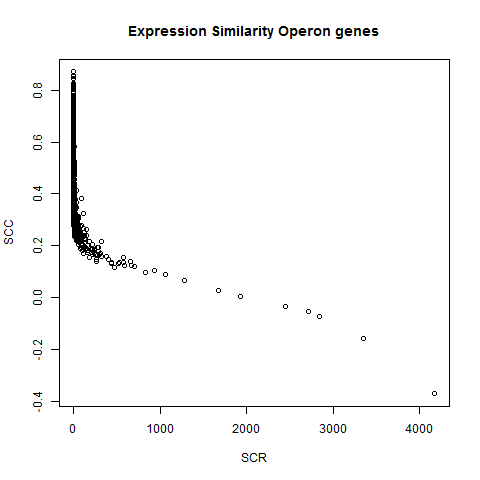


***Figure S2*** *This figure displays for the positive control i.e. operonic genes that are expected to be well coexpressed, their pairwise Spearman Correlation Coefficient or SCC (Y-axis) as a function of their pairwise Spearman Correlation Rank or SCR (rank-based derivative of SCC) (X-axis). It shows that for most pairs of within operonic genes, their coexpression degree as measured by the SCR is generally higher (low SCR meaning high coexpression degree) than their coexpression degree assessed by the SCC.*

**3. The degree of coexpression of genes that are coregulated in *E. coli* is generally low**

In general it is assumed that genes that are coregulated by the same Transcription Factors (TFs) tend to be highly coexpressed [2]. To have an intuition of the absolute degree of coexpression of coregulated genes in *E. coli* we compared their coexpression with that of genes that are located in the same transcription unit (operonic genes) and that thus should display the maximal levels of coexpression.

To this end we evaluated the degree of coexpression genes located (1) in the same operon, versus the degree of coexpression of genes that are (2) coregulated but not within the same operon (definitions of operons and coregulated genes are described in Materials and Methods).

The degree of coexpression of operonic genes and coregulated genes as measured by SCR was shown in Fig S2 by boxplotting the SCR values for respectively operonic (left panel) and coregulated genes (right panel).
Operonic genes were mostly highly coexpressed (low SCR), while the majority of coregulated non-operonic genes displayed much lower degrees of coexpression (high SCR).


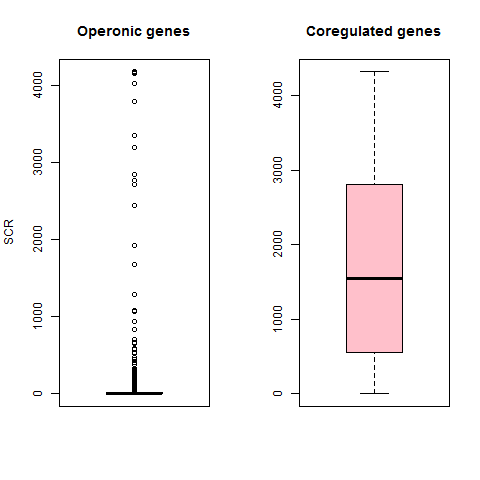


***Fig S2. Coexpression of genes within operons and of coregulated genes in E. coli****. Coexpression degrees of operonic genes and coregulated genes are shown by boxplots of SCR values of gene pairs extracted from respectively operons (left panel) and of gene pairs coregulated by at least one TF (right panel).*

**4. Evidence classification of interactions in RegulonDB**

RegulonDB distinguishes between TF-gene interactions supported by strong versus weak evidence. Interactions are classified as ‘based on strong evidence’ if they are supported by at least one source of strong evidence and ‘based on weak evidence’ if they are supported by weak evidence only. According to RegulonDB, “**Weak evidence** is a single evidence with more ambiguous conclusions, where alternative explanations, indirect effects, or potential false positives are prevalent, as well as computational predictions; for instance gel mobility shift assays with cell extracts or gene expression analysis and **Strong evidence** is a single evidence with direct physical interaction or solid genetic evidence with a low probability for alternative explanations; for instance, footprinting with purified protein or site mutation.”

To ensure that the main conclusions of our analyses were not influenced by whether or not we included interactions with weak evidence, we tested the impact of using different sets of interactions on our result, more specifically we tested:

- a set including **all interactions** supported by both weak or strong evidence (i.e. 3430 interactions corresponding to 98795 coregulated gene pairs)

- a **partially reduced set of interactions** excluding interactions supported by at most one type of weak evidence only (i.e. 2961 interactions corresponding to 78772 coregulated gene pairs or 86% of the number of coregulated gene pairs of the full set of coregulated gene pairs)

- a set of **strongly evidenced interactions only** containing interactions based on strong evidence only - in comparison to the previous setting here also interactions that are supported by two types of weak evidence are excluded (i.e. 2213 interactions corresponding to 30894 coregulated gene pairs or 31% of the full set of coregulated gene pairs).

We redid the analysis represented in the main text with each of the datasets mentioned above (all interactions, partially reduced dataset and the dataset containing strongly evidenced interactions only). Fig S3 represents the effect of the distance on the degree of coexpression as obtained for each of the datasets. Overall tendencies were similar, irrespective of the dataset that was used. Except for the case where genes are ‘coregulated by 1 TF with complete overlap of regulatory programs’ the tendency observed for the effect of the distance on the degree of coexpression was different between the results obtained for the different datasets and especially non-monotonic for the most stringent dataset (only interactions supported by strong evidence, blue dotted curve, right lower panel). Because of the non-monotonic behavior in case of the most stringent condition, we believe that in this setting the dataset becomes too small to observe a consistent behavior (this dataset contained 1046 pairs of genes instead of 1461 pairs of genes in case of the partially reduced dataset).

Results thus show that in general conclusions are not affected by including weak interactions. As the partially reduced dataset offers the best trade-off between using high confidence interactions and still offering sufficient data to observe tendencies, all the results in the main text were obtained with this dataset.


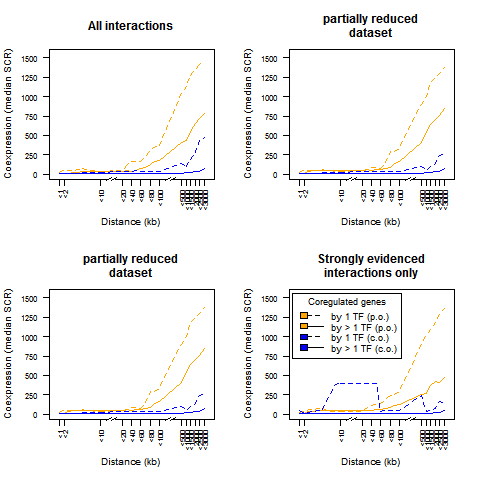


Fig S3. ***Effect of coregulation tightness and of the distance between coregulated genes on the coexpression degree for different types of datasets****. Left upper panel: all interactions, right upper and left lower panel: partially reduced dataset, right lower panel: strongly supported interactions only. The coexpression behavior of coregulated genes was disentangled, depending on whether the regulatory programs displayed complete overlap (c.o.) versus partial overlap (p.o.) (blue versus orange) and depending on the number of common TFs present in the overlapping part of their regulatory program (dotted line for 1 TF versus full line for >1 TF).*

**5. Evidence of horizontal gene co-transfer in genes with strong distance conservation**

As explained in the results we found indications that for highly coexpressed genes located in each other’s neighborhood on the genome there is an evolutionary constraint on conserving their small distance. Because evolutionary conservation of close distance of genes has been associated with horizontal gene co-transfer we evaluated whether highly coexpressed genes that are nearby located and that have strong distance conservation show evidence of horizontal gene co-transfer.

Hereto we selected cases of coregulated genes that were located at small distances (< 5 intervening genes), that were highly coexpressed (SCR < 100), which had an orthologous counterpart in most other species (> 90% of gamma-proteobacteria) and for which the intergenic distance was highly conserved (distance conservation > 0.4). This resulted in 75 pairs of nearby located coregulated genes. We then assessed whether these pairs of genes were co-acquired by horizontal gene co-transfer.

We found that these 75 pairs of genes belonged to 12 different pairs of operons of which 9 show evidence of horizontal operon co-transfer: *araBAD-araC*, whose genes are co-transferred as found repeatedly within y-Proteobacteria; *csgBAC-csgDEFG* and *narGHJI-narK* [3]; *rhaSR-rhaBAD*, which shows ancestral co-transfer; and *cusRS-cusCFBA*, a more recent co-transfer within the *E. coli - Shigella* lineage [4]. Of the remaining operons we did not find any evidence in the literature of HGT, nor did we find support for their HGT in computational predictions [5].

This indicates that for highly coexpressed nearby located genes there exists an evolutionary constraint for maintaining their small distance.

**REFERENCES**

1. Adhya S, Gottesman M. Control of transcription termination. Annu Rev Biochem. 1978;47: 967–96. doi:10.1146/annurev.bi.47.070178.004535

2. Yu H, Luscombe NM, Qian J, Gerstein M. Genomic analysis of gene expression relationships in transcriptional regulatory networks. Trends Genet. 2003;19: 422–427. doi:http://dx.doi.org/10.1016/S0168-9525(03)00175-6

3. Price MN, Dehal PS, Arkin AP. Horizontal gene transfer and the evolution of transcriptional regulation in Escherichia coli. Genome Biol. BioMed Central; 2008;9: R4–R4. doi:10.1186/gb-2008-9-1-r4

4. Skippington E, Ragan MA. Within-species lateral genetic transfer and the evolution of transcriptional regulation in Escherichia coli and Shigella. BMC Genomics. BioMed Central; 2011;12: 532. doi:10.1186/1471-2164-12-532

5. Garcia-Vallve S, Guzman E, Montero MA, Romeu A. HGT-DB: a database of putative horizontally transferred genes in prokaryotic complete genomes. Nucleic Acids Res. Oxford, UK: Oxford University Press; 2003;31: 187–189. Available: http://www.ncbi.nlm.nih.gov/pmc/articles/PMC165451/
