# Supplementary material for: Effect of genomic distance on coexpression of coregulated genes in E. coli
Source: PLoS One. 2017 Apr 18;12(4):e0174887. doi: 10.1371/journal.pone.0174887 (PMC5395161; doi:10.1371/journal.pone.0174887)
Supplement: S1 Table — This table shows all TFs considered in our analysis with at least one pair of coregulated genes (see Materials and methods for the definition of coregulated genes). For each TF we showed the number of pairs of genes coregulated by that TF, the mean distance between every two genes in a pair (in base pairs), and the mean coexpression (as measured by SCR). (DOC) [file pone.0174887.s002.doc]

| TF | Number of gene pairs coregulated by this TF | Mean Distance between genes in a pair (base pairs) | Mean Coexpression of all gene pairs coregulated by this TF (SCR) |
| --- | --- | --- | --- |
| AcrR | 2 | 750 | 24 |
| Ada | 5 | 1359698 | 450 |
| AdiY | 6 | 1522945 | 1176 |
| AgaR | 34 | 3566 | 1088 |
| AllR | 8 | 5193 | 364 |
| AraC | 30 | 1441575 | 604 |
| ArgP | 56 | 1704110 | 1481 |
| ArgR | 373 | 1650688 | 677 |
| AscG | 5 | 1962405 | 1312 |
| BaeR | 6 | 426382 | 592 |
| BasR | 22 | 2081042 | 1806 |
| BetI | 3 | 1025 | 15 |
| BirA | 4 | 1622 | 5 |
| CaiF | 24 | 5344 | 61 |
| CpxR | 274 | 1650091 | 1888 |
| Cra | 1074 | 1636648 | 1927 |
| CsgD | 26 | 1210469 | 1175 |
| CueR | 1 | 369466 | 1 |
| CusR | 8 | 2124 | 19 |
| CysB | 11 | 264253 | 13 |
| CytR | 16 | 1255697 | 1537 |
| DcuR | 28 | 1963434 | 1494 |
| DnaA | 20 | 1405114 | 1515 |
| DpiA | 28 | 1522678 | 1791 |
| EvgA | 31 | 688546 | 1466 |
| FadR | 10 | 966395 | 493 |
| FhlA | 101 | 250116 | 165 |
| FlhDC | 308 | 686602 | 321 |
| FliZ | 72 | 1686520 | 1610 |
| Fur | 2136 | 1799345 | 1742 |
| GadE | 69 | 728648 | 1346 |
| GadE-RcsB | 10 | 344825 | 845 |
| GadX | 19 | 664042 | 16 |
| GalR | 13 | 1614163 | 1567 |
| GalS | 20 | 1327261 | 490 |
| GcvA | 3 | 105733 | 3073 |
| GlpR | 23 | 1483701 | 15 |
| GntR | 33 | 1494359 | 1737 |
| IclR | 3 | 4196 | 2988 |
| IdnR | 4 | 1768 | 45 |
| IlvY | 1 | 150 | 2063 |
| IscR | 106 | 1198371 | 3056 |
| LeuO | 7 | 2580480 | 516 |
| LexA | 166 | 1744735 | 1375 |
| LrhA | 1 | 2135397 | 847 |
| Lrp | 516 | 1769136 | 1597 |
| LsrR | 12 | 3726 | 8 |
| MalI | 2 | 976 | 104 |
| MalT | 29 | 406035 | 10 |
| MarA | 105 | 897553 | 1475 |
| MatA | 1 | 75 | 47 |
| MelR | 4 | 1012 | 42 |
| MetJ | 51 | 2036143 | 163 |
| MetR | 6 | 989591 | 1471 |
| Mlc | 23 | 770364 | 202 |
| MlrA | 14 | 2351197 | 2553 |
| MngR | 2 | 1106 | 1009 |
| MntR | 1 | 1661356 | 701 |
| ModE | 346 | 1244177 | 1393 |
| MqsA | 3 | 1495974 | 283 |
| Nac | 13 | 2050874 | 2480 |
| NadR | 2 | 1925712 | 2 |
| NagC | 136 | 1663846 | 1909 |
| NanR | 8 | 1166339 | 777 |
| NarL | 1280 | 1560620 | 1053 |
| NarP | 384 | 1308849 | 1144 |
| NhaR | 14 | 962683 | 1171 |
| NrdR | 20 | 1267052 | 1721 |
| NsrR | 1167 | 1551150 | 2149 |
| NtrC | 199 | 1308916 | 687 |
| OmpR | 7 | 1069945 | 2193 |
| OxyR | 56 | 1391792 | 1822 |
| PaaX | 11 | 4051 | 157 |
| PdhR | 314 | 1386884 | 2054 |
| PepA | 2 | 4450044 | 843 |
| PhoB | 515 | 2277882 | 1891 |
| PhoP | 326 | 1724125 | 2227 |
| PspF | 5 | 2893484 | 768 |
| PurR | 361 | 1507021 | 211 |
| PutA | 1 | 423 | 2 |
| PuuR | 17 | 2526 | 9 |
| RcdA | 4 | 567790 | 2187 |
| RcnR | 1 | 121 | 347 |
| RcsB | 33 | 732146 | 865 |
| RhaS | 11 | 2312 | 55 |
| Rob | 14 | 934211 | 899 |
| RstA | 4 | 114833 | 1031 |
| RutR | 11 | 1909293 | 2293 |
| SoxS | 153 | 1662221 | 1359 |
| TrpR | 5 | 2013247 | 167 |
| TyrR | 10 | 1447247 | 601 |
