# Supplementary material for: Effect of genomic distance on coexpression of coregulated genes in E. coli
Source: PLoS One. 2017 Apr 18;12(4):e0174887. doi: 10.1371/journal.pone.0174887 (PMC5395161; doi:10.1371/journal.pone.0174887)
Supplement: S2 Table — This table shows an excerpt of pairs of genes that belong to the negative control of genes not known to be coregulated but highly coexpressed and located nearby and that were predicted to be coregulated according to SELEX. Gene 1 and gene 2 correspond to a pair of genes selected from the set of so-called non-coregulated genes with selection criteria 1) small distance (< 10 kb) 2) high degree of coexpression (SCR<10) and 3) at least one common TF in their respective set of TFs as predicted by SELEX. The coexpression degree between gene 1 and gene 2 is given in SCR. One or more predicted common TF(s) was (were) given. The numbers between brackets refer to the Nth best hit (which means Nth highest % similarity of that TF for that gene) that TF was for respectively gene 1 and gene 2. (DOCX) [file pone.0174887.s003.docx]

| TF | Gene 1 | Gene 2 | Distance between gene 1 and gene 2 (bps) | Transcriptional role of TF on both genes (r = corepressed, a = coactivated) | Coexpression value of gene 1 and gene 2 (SCR) |
| --- | --- | --- | --- | --- | --- |
| AcrR | acrA | acrR | 142 | r | 12 |
| AcrR | acrB | acrR | 1358 | r | 35 |
| Ada | alkA | alkB | 161149 | a | 892 |
| Ada | alkA | aidB | 2266734 | a | 957 |
| Ada | alkA | ada | 161799 | a | 48 |
| Ada | alkB | aidB | 2104935 | a | 220 |
| Ada | aidB | ada | 2103871 | a | 131 |
| AdiY | gadC | adiA | 2767764 | a | 232 |
| AdiY | gltB | adiA | 979070 | a | 2668 |
| AdiY | gadA | adiA | 670674 | a | 122 |
| AdiY | gadB | adiA | 2766208 | a | 209 |
| AdiY | gltD | adiA | 977639 | a | 611 |
| AgaR | agaI | agaW | 5168 | r | 346 |
| AgaR | kbaY | agaA | 1518 | r | 25 |
| AgaR | agaV | agaI | 5580 | r | 1701 |
| AgaR | agaR | agaI | 7605 | r | 3409 |
| AgaR | agaC | agaA | 3060 | r | 13 |
| AllR | allS | hyi | 3489 | r | 378 |
| AllR | allA | allB | 6214 | r | 1046 |
| AllR | glxR | allS | 4365 | r | 47 |
| AllR | allS | allB | 6926 | r | 492 |
| AllR | allS | allA | 230 | r | 9 |
| AlsR | rpiR | rpiB | 359 | r | 2 |
| AlsR | rpiB | alsA | 2370 | r | 11 |
| AlsR | rpiB | alsC | 3881 | r | 151 |
| AlsR | rpiB | alsE | 4872 | r | 515 |
| AlsR | rpiB | alsB | 1308 | r | 1 |
| AppY | appC | hyaA | 4483 | a | 3 |
| AppY | appC | hyaF | 134 | a | 7 |
| AppY | appB | hyaD | 2939 | a | 8 |
| AppY | appC | hyaC | 1967 | a | 6 |
| AppY | appB | hyaF | 1690 | a | 8 |
| AraC | araC | araE | 2907521 | a | 1790 |
| AraC | araD | araE | 2912236 | a | 1088 |
| AraC | araA | araH | 1912241 | a | 394 |
| AraC | araG | araB | 1911531 | a | 17 |
| AraC | araA | araJ | 342184 | a | 1237 |
| ArgP | lysC | dapD | 4043960 | a | 33 |
| ArgP | lysA | argO | 89274 | a | 453 |
| ArgP | argO | dapD | 2880248 | a | 1068 |
| ArgP | lysA | lysC | 1252986 | a | 2 |
| ArgP | dnaA | lysC | 348155 | a | 1542 |
| ArgR | rpsO | argH | 845167 | r | 425 |
| ArgR | gltB | artP | 2449697 | r | 3542 |
| ArgR | hisJ | argD | 1062172 | r | 1286 |
| ArgR | hisP | argD | 1064451 | r | 2854 |
| ArgR | artM | hisQ | 1522495 | r | 33 |
| AscG | prpR | ascB | 2491345 | r | 1395 |
| AscG | prpR | ascF | 2489879 | r | 454 |
| AscG | prpR | htpG | 146677 | r | 892 |
| AscG | ascB | htpG | 2342794 | r | 1725 |
| AscG | ascF | htpG | 2341328 | r | 2093 |
| BaeR | mdtB | acrD | 429208 | a | 17 |
| BaeR | acrD | mdtC | 426130 | a | 2 |
| BaeR | mdtD | acrD | 424714 | a | 473 |
| BaeR | acrD | baeR | 422595 | a | 2187 |
| BaeR | mdtA | acrD | 432330 | a | 306 |
| BasR | csgF | dgkA | 3153310 | a | 4176 |
| BasR | hha | dgkA | 3775128 | a | 1405 |
| BasR | cspI | csgD | 534060 | a | 200 |
| BasR | cspI | csgF | 535129 | a | 1172 |
| BasR | cspI | fimB | 2902289 | a | 637 |
| BetI | betB | betT | 730 | r | 34 |
| BetI | betI | betT | 129 | r | 3 |
| BetI | betA | betT | 2216 | r | 6 |
| BirA | bioF | bioA | 1124 | r | 2 |
| BirA | bioC | bioA | 2265 | r | 5 |
| BirA | bioD | bioA | 3013 | r | 13 |
| BirA | bioA | bioB | 87 | r | 2 |
| CadC | cadB | cadC | 365 | a | 11 |
| CadC | cadA | cadC | 1779 | a | 4 |
| CaiF | caiA | fixC | 3794 | a | 130 |
| CaiF | caiB | fixA | 3288 | a | 54 |
| CaiF | caiA | fixA | 2017 | a | 87 |
| CaiF | fixX | caiE | 10092 | a | 33 |
| CaiF | caiT | fixC | 2249 | a | 81 |
| CdaR | garL | garD | 1725 | a | 2 |
| CdaR | garK | gudP | 348525 | a | 477 |
| CdaR | garP | gudD | 354188 | a | 52 |
| CdaR | garL | gudX | 352041 | a | 147 |
| CdaR | garR | garD | 2525 | a | 14 |
| CpxR | ftnB | aroG | 1199041 | a | 1573 |
| CpxR | dsbA | lpxD | 3839445 | a | 821 |
| CpxR | baeS | ftnB | 175448 | a | 3605 |
| CpxR | baeR | lpxD | 1960304 | a | 3206 |
| CpxR | cheA | ompF | 985179 | r | 64 |
| Cra | lpd | prpB | 218570 | r | 3668 |
| Cra | pdhR | mtlD | 3649591 | r | 1806 |
| Cra | fbaB | edd | 242906 | r | 3767 |
| Cra | aroP | crr | 2412305 | a | 1746 |
| Cra | envC | manX | 1883818 | r | 3609 |
| CsgD | nlpA | adrA | 3433156 | a | 3773 |
| CsgD | pepD | fliK | 1760694 | r | 2207 |
| CsgD | adrA | csgE | 697333 | a | 1579 |
| CsgD | pepD | fliG | 1757188 | r | 3450 |
| CsgD | csgD | nlpA | 2734779 | a | 195 |
| CspA | hns | gyrA | 1042670 | a | 4027 |
| CueR | copA | cueO | 369466 | a | 1 |
| CusR | cusR | cusA | 3271 | a | 6 |
| CusR | cusA | cusS | 3944 | a | 103 |
| CusR | cusB | cusS | 2709 | a | 11 |
| CusR | cusS | cusC | 830 | a | 5 |
| CusR | cusR | cusC | 157 | a | 5 |
| CysB | cysC | cysW | 331708 | a | 12 |
| CysB | cysK | cysD | 342041 | a | 5 |
| CysB | cysD | cysP | 331893 | a | 2 |
| CysB | cbl | cysP | 481596 | a | 11 |
| CysB | cysH | cysC | 13586 | a | 4 |
| CytR | deoC | cytR | 492867 | r | 442 |
| CytR | cytR | deoA | 493773 | r | 2557 |
| CytR | ppiA | cytR | 631135 | r | 40 |
| CytR | rpoH | cytR | 522648 | r | 3464 |
| CytR | cdd | cytR | 1890704 | r | 1272 |
| DcuR | dpiA | dctA | 3026419 | a | 4295 |
| DcuR | frdC | dctA | 695930 | a | 178 |
| DcuR | dpiA | frdD | 3723265 | a | 1830 |
| DcuR | frdA | dctA | 697063 | a | 281 |
| DcuR | frdC | dcuB | 30633 | a | 20 |
| DicA | intQ | dicC | 3662 | r | 200 |
| DicA | intQ | insD | -25 | r | 855 |
| DicA | dicB | dicC | 1759 | r | 129 |
| DicA | dicB | insD | 1084 | r | 142 |
| DicA | dicC | insD | 3031 | r | 2133 |
| DinJ | cspE | dinJ | 410013 | r | 1251 |
| DnaA | dnaA | aldA | 2392654 | r | 3618 |
| DnaA | recF | guaA | 1247614 | r | 811 |
| DnaA | recF | aldA | 2390476 | r | 4060 |
| DnaA | recF | rpoH | 279365 | r | 2145 |
| DnaA | recF | guaB | 1246079 | r | 860 |
| DpiA | mdh | citX | 2734094 | a | 2509 |
| DpiA | citF | dpiA | 4291 | a | 672 |
| DpiA | exuT | citE | 2593413 | a | 1228 |
| DpiA | exuT | citX | 2595868 | a | 983 |
| DpiA | dpiB | citC | 379 | a | 1575 |
| EnvY | ompF | ompC | 1323463 | a | 1601 |
| EvgA | emrK | gadE | 1175028 | a | 1121 |
| EvgA | evgS | mdtE | 1171266 | a | 1097 |
| EvgA | emrK | mdtE | 1175894 | a | 959 |
| EvgA | emrY | mdtF | 1178239 | a | 1010 |
| EvgA | emrK | acrD | 104256 | a | 2553 |
| FadR | fadR | fabA | 218468 | a | 313 |
| FadR | fadR | fabB | 1203527 | a | 548 |
| FadR | fadJ | fadD | 567267 | r | 30 |
| FadR | fabB | fabA | 1422714 | a | 498 |
| FadR | iclR | fabA | 3205134 | a | 1520 |
| FhlA | hycF | hypD | 7384 | a | 184 |
| FhlA | hypF | hyfG | 225019 | a | 153 |
| FhlA | hycI | hypA | 7604 | a | 18 |
| FhlA | hyfD | hycG | 237193 | a | 2564 |
| FhlA | hyfH | hycB | 238529 | a | 533 |
| FlhDC | recC | nrfG | 1331116 | a | 2125 |
| FlhDC | ccmB | fliH | 279806 | a | 715 |
| FlhDC | ccmD | ppdC | 666855 | a | 182 |
| FlhDC | ccmA | fliM | 275928 | a | 2480 |
| FlhDC | fliP | ccmF | 270353 | a | 1409 |
| FliZ | csgG | mlrA | 1111981 | r | 502 |
| FliZ | csgA | hdeA | 2550306 | r | 196 |
| FliZ | csgB | gadC | 463349 | r | 236 |
| FliZ | mlrA | hdeB | 1440370 | r | 144 |
| FliZ | mdtF | csgE | 2556673 | r | 1211 |
| FucR | fucP | fucA | 547 | a | 4 |
| FucR | fucK | fucA | 3750 | a | 9 |
| FucR | fucR | fucA | 5680 | a | 68 |
| FucR | fucU | fucO | 5875 | a | 76 |
| FucR | fucI | fucA | 1896 | a | 16 |
| Fur | cyoB | fepA | 159612 | r | 226 |
| Fur | entD | nrdH | 2189443 | r | 93 |
| Fur | rcnA | nrdH | 613982 | r | 91 |
| Fur | pyrC | nrdI | 1677157 | r | 4186 |
| Fur | ndh | exbB | 1982660 | r | 1320 |
| GadE | lpxD | cyoC | 245274 | a | 824 |
| GadE | gadX | gnd | 1563717 | a | 1811 |
| GadE | mdtE | hdeA | 2492 | a | 13 |
| GadE | hdeA | gadW | 7150 | a | 17 |
| GadE | cyoA | fabZ | 247331 | a | 440 |
| GadE-RcsB | aslB | cadB | 374504 | a | 537 |
| GadE-RcsB | adiC | gadA | 668114 | a | 36 |
| GadE-RcsB | gadX | aslB | 317148 | a | 3200 |
| GadE-RcsB | adiC | cadA | 19439 | a | 37 |
| GadE-RcsB | adiC | cadB | 21666 | a | 7 |
| GadW | dctR | gadW | 8677 | r | 9 |
| GadX | asnB | amtB | 223260 | a | 300 |
| GadX | mdtE | dctR | 4019 | a | 6 |
| GadX | rpoS | gadW | 796340 | a | 480 |
| GadX | gadE | gadA | 7287 | a | 4 |
| GadX | rpoS | amtB | 2391105 | a | 1798 |
| GalR | galR | galT | 2184369 | r | 2812 |
| GalR | galS | galR | 734931 | r | 1470 |
| GalR | galP | galE | 2295028 | r | 78 |
| GalR | galM | galP | 2298246 | r | 221 |
| GalR | galM | galR | 2186561 | r | 933 |
| GalS | mglC | galT | 1444513 | r | 119 |
| GalS | galE | mglB | 1446094 | r | 43 |
| GalS | mglC | galP | 850531 | r | 1104 |
| GalS | mglA | galK | 1446589 | r | 39 |
| GalS | galP | galS | 846616 | r | 779 |
| GcvA | gcvA | gcvT | 107006 | r | 2577 |
| GcvA | gcvA | gcvH | 106593 | r | 3904 |
| GcvA | gcvA | gcvP | 103601 | r | 2739 |
| GlpR | glpQ | glpK | 1764704 | r | 4 |
| GlpR | glpA | glpK | 1761440 | r | 5 |
| GlpR | glpC | glpK | 1759004 | r | 8 |
| GlpR | glpT | glpD | 1209640 | r | 14 |
| GlpR | glpX | glpT | 1762196 | r | 41 |
| GntR | gntK | idnD | 915783 | r | 2178 |
| GntR | nfuA | idnK | 948425 | r | 1723 |
| GntR | gntT | gntU | 27847 | r | 4 |
| GntR | gntK | idnK | 917031 | r | 682 |
| GntR | idnD | gntX | 947811 | r | 2208 |
| HU | mtr | galE | 2511317 | r | 4240 |
| HU | mtr | pgm | 2588174 | r | 3801 |
| HU | mtr | galT | 2512343 | r | 4205 |
| HU | pgm | galT | 74785 | r | 609 |
| HU | mtr | galM | 2514535 | r | 2182 |
| IclR | iclR | aceA | 4391 | r | 3442 |
| IclR | iclR | aceK | 2472 | r | 2043 |
| IclR | iclR | aceB | 5725 | r | 3478 |
| IdnR | idnT | idnK | 2098 | a | 51 |
| IdnR | idnK | idnD | 217 | a | 2 |
| IdnR | idnK | idnO | 1272 | a | 7 |
| IdnR | idnK | idnR | 3484 | a | 120 |
| IlvY | ilvC | ilvY | 150 | r | 2063 |
| IscR | iscA | napF | 356066 | r | 4173 |
| IscR | erpA | iscR | 2482711 | r | 7 |
| IscR | hyaF | nfuA | 2506817 | r | 2857 |
| IscR | iscU | erpA | 2480971 | r | 18 |
| IscR | iscR | napF | 358146 | r | 4311 |
| LeuO | bglJ | leuA | 4518654 | a | 552 |
| LeuO | bglF | leuC | 3820879 | a | 1603 |
| LeuO | bglB | leuB | 3818354 | a | 2641 |
| LeuO | bglJ | leuD | 4522730 | a | 977 |
| LeuO | leuC | bglJ | 4521319 | a | 1846 |
| LexA | murF | dinG | 736285 | r | 392 |
| LexA | dinJ | ftsQ | 142257 | r | 2905 |
| LexA | ftsZ | dinF | 4149309 | r | 3860 |
| LexA | phr | dinJ | 492228 | r | 490 |
| LexA | recX | ftsZ | 2713705 | r | 3882 |
| LrhA | fimE | lrhA | 2135397 | a | 847 |
| Lrp | adhE | ompC | 1012324 | r | 73 |
| Lrp | livJ | oppB | 2294735 | r | 3830 |
| Lrp | ilvD | oppD | 2647710 | r | 2086 |
| Lrp | fimF | ilvH | 4457917 | a | 2181 |
| Lrp | adhE | ilvE | 2653163 | r | 1483 |
| LsrR | lsrF | lsrR | 4859 | r | 4 |
| LsrR | lsrF | lsrK | 5891 | r | 1 |
| LsrR | lsrR | lsrA | 249 | r | 5 |
| LsrR | lsrR | lsrD | 2806 | r | 13 |
| LsrR | lsrB | lsrK | 4842 | r | 3 |
| MalI | malX | malI | 175 | r | 127 |
| MalI | malY | malI | 1777 | r | 80 |
| MalT | malK | malZ | 3821251 | a | 736 |
| MalT | malE | malM | 3135 | a | 2 |
| MalT | malG | lamB | 4455 | a | 4 |
| MalT | malE | malP | 692757 | a | 4 |
| MalT | malS | malZ | 3311964 | a | 205 |
| MarA | putA | pqiA | 61666 | a | 3488 |
| MarA | acrB | pqiB | 528855 | a | 420 |
| MarA | putA | nfo | 1170757 | a | 2745 |
| MarA | pqiB | nfsB | 407835 | a | 2180 |
| MarA | marA | poxB | 707326 | a | 2840 |
| MatA | ecpR | ecpA | 75 | a | 47 |
| MelR | melB | melR | 1741 | a | 52 |
| MelR | melA | melR | 283 | a | 32 |
| MelR | melB | melR | 1741 | r | 52 |
| MelR | melR | melA | 283 | r | 32 |
| MetJ | metI | metR | 3788265 | r | 3 |
| MetJ | ahpF | metB | 3486154 | r | 1912 |
| MetJ | ahpF | metE | 3370535 | r | 1751 |
| MetJ | metK | metR | 924004 | r | 21 |
| MetJ | metR | metQ | 3788958 | r | 31 |
| MetR | glyA | hmp | 328 | a | 3118 |
| MetR | metH | glyA | 1538322 | a | 205 |
| MetR | metH | metE | 208514 | a | 3 |
| MetR | glyA | metE | 1327547 | a | 47 |
| MetR | metE | hmp | 1326029 | a | 3565 |
| Mlc | ptsG | manZ | 743394 | r | 18 |
| Mlc | ptsI | mlc | 865500 | r | 605 |
| Mlc | ptsH | manZ | 629016 | r | 158 |
| Mlc | manZ | crr | 631086 | r | 168 |
| Mlc | ptsG | mlc | 506843 | r | 199 |
| MlrA | rpmA | csgD | 2228465 | a | 3108 |
| MlrA | rpmA | csgF | 2229534 | a | 2880 |
| MlrA | rplU | csgD | 2228743 | a | 2643 |
| MlrA | cadC | csgF | 3257069 | a | 871 |
| MlrA | rplU | cadC | 1026946 | a | 1682 |
| MngR | mngA | mngR | 109 | r | 1925 |
| MngR | mngB | mngR | 2103 | r | 92 |
| MntR | mntH | dps | 1661356 | r | 701 |
| ModE | oppA | deoD | 3318069 | r | 682 |
| ModE | napF | hycI | 539076 | a | 1349 |
| ModE | hycE | napC | 546503 | a | 2593 |
| ModE | napF | moaB | 1483235 | a | 2606 |
| ModE | modB | oppC | 506084 | r | 69 |
| MprA | nmpC | acrB | 91354 | r | 1712 |
| MprA | nmpC | mprA | 2232744 | r | 703 |
| MprA | nmpC | emrB | 2234590 | r | 2447 |
| MprA | emrA | acrA | 2324606 | r | 149 |
| MprA | acrB | emrA | 2325822 | r | 101 |
| MqsA | cspD | rpoS | 1942768 | r | 29 |
| MqsA | cspD | mqsR | 2244457 | r | 182 |
| MqsA | rpoS | mqsR | 300697 | r | 637 |
| Nac | gabT | codB | 2435352 | a | 3900 |
| Nac | mioC | nac | 1864078 | r | 1667 |
| Nac | gltD | nac | 1297263 | r | 908 |
| Nac | nac | gdhA | 217302 | r | 403 |
| Nac | nupC | codA | 2154386 | a | 272 |
| NadR | pncB | nadA | 206026 | r | 33 |
| NadR | pncB | pnuC | 205269 | r | 38 |
| NadR | pncB | nadB | 1718863 | r | 128 |
| NadR | nadA | nadB | 1926091 | r | 1 |
| NadR | pnuC | nadB | 1925334 | r | 3 |
| NagC | crr | nagC | 1833039 | r | 2425 |
| NagC | chbC | nanM | 2716444 | r | 736 |
| NagC | chbC | nagE | 1112767 | r | 44 |
| NagC | umpH | glmS | 3210313 | r | 462 |
| NagC | creB | manZ | 2731260 | r | 791 |
| NanR | nanA | nanM | 1164084 | r | 1732 |
| NanR | nanM | nanE | 1166624 | r | 1330 |
| NanR | nanK | nanC | 1168436 | r | 510 |
| NanR | nanC | nanE | 1167750 | r | 787 |
| NanR | nanM | nanT | 1165086 | r | 661 |
| NarL | nirC | cydC | 2566607 | a | 2895 |
| NarL | aspA | norW | 1531847 | r | 3901 |
| NarL | hybA | ccmG | 851190 | r | 286 |
| NarL | hybB | adhE | 1843664 | r | 667 |
| NarL | citD | hybD | 2488808 | r | 285 |
| NarP | fdnH | ccmB | 745015 | r | 80 |
| NarP | ccmG | nirB | 1201047 | a | 1612 |
| NarP | ccmG | hyaA | 1257949 | r | 1592 |
| NarP | napG | fdhF | 1996960 | a | 2281 |
| NarP | napG | hyaF | 1260758 | r | 1390 |
| NhaR | pgaB | osmC | 465569 | a | 1280 |
| NhaR | pgaB | nhaR | 1067442 | a | 520 |
| NhaR | pgaA | osmC | 463137 | a | 1445 |
| NhaR | nhaA | pgaD | 1066674 | a | 902 |
| NhaR | pgaA | nhaA | 1070434 | a | 1497 |
| NrdR | nrdI | nrdA | 453815 | r | 257 |
| NrdR | nrdH | nrdG | 1658933 | r | 3497 |
| NrdR | nrdD | nrdI | 1659148 | r | 2296 |
| NrdR | nrdB | nrdE | 452834 | r | 927 |
| NrdR | nrdE | nrdA | 454198 | r | 73 |
| NsrR | dkgB | rffM | 3748009 | r | 729 |
| NsrR | hycE | rffH | 1127138 | r | 503 |
| NsrR | nrfE | aceE | 4163855 | r | 4136 |
| NsrR | rffA | hcr | 3061796 | r | 2461 |
| NsrR | nrfD | aceE | 4162819 | r | 4136 |
| NtrC | hisJ | ddpA | 863523 | a | 19 |
| NtrC | glnQ | glnL | 3207627 | a | 69 |
| NtrC | astB | ddpC | 267006 | a | 12 |
| NtrC | ddpX | hisM | 861439 | a | 52 |
| NtrC | hisJ | nac | 364071 | a | 329 |
| OmpR | ompC | csgF | 1208318 | a | 1056 |
| OmpR | fadL | ecnB | 1913908 | r | 2559 |
| OmpR | csgE | ompC | 1207904 | a | 1332 |
| OmpR | nmpC | ecnB | 3798528 | r | 4287 |
| OmpR | bolA | flhC | 1521277 | r | 4077 |
| OxyR | ahpF | dsbG | 1180 | a | 1200 |
| OxyR | fur | ahpC | 70692 | a | 499 |
| OxyR | gor | katG | 486184 | a | 470 |
| OxyR | fur | hcp | 201516 | a | 2962 |
| OxyR | hemH | katG | 3633617 | a | 3244 |
| PaaX | paaD | paaZ | 2283 | r | 2 |
| PaaX | paaZ | paaB | 1226 | r | 177 |
| PaaX | paaG | paaZ | 4622 | r | 11 |
| PaaX | paaZ | paaF | 3855 | r | 17 |
| PaaX | paaZ | paaA | 285 | r | 7 |
| PdhR | murD | cyoC | 348867 | r | 1333 |
| PdhR | ddlB | cyoA | 346734 | r | 1603 |
| PdhR | pdhR | murG | 21381 | r | 254 |
| PdhR | ftsI | cyoE | 352860 | r | 2748 |
| PdhR | hha | ddlB | 376161 | r | 3275 |
| PepA | carB | pepA | 4448425 | r | 1127 |
| PepA | carA | pepA | 4451664 | r | 558 |
| PhoB | pstA | gadW | 243931 | a | 2511 |
| PhoB | pstS | psiE | 328800 | a | 820 |
| PhoB | phnD | asr | 2651651 | a | 1714 |
| PhoB | phnJ | phoU | 411183 | a | 1602 |
| PhoB | phnD | phoU | 415758 | a | 2411 |
| PhoP | gadE | ompT | 3071533 | a | 2985 |
| PhoP | metL | glgP | 563254 | a | 2852 |
| PhoP | iraM | mgrB | 695421 | a | 448 |
| PhoP | phoQ | hdeD | 2466019 | a | 2718 |
| PhoP | glgA | hemL | 3389741 | a | 3556 |
| PspF | pspD | pspG | 2893225 | a | 94 |
| PspF | pspB | pspG | 2893814 | a | 54 |
| PspF | pspC | pspG | 2893455 | a | 429 |
| PspF | pspE | pspG | 2892836 | a | 3054 |
| PspF | pspA | pspG | 2894092 | a | 208 |
| PurR | codB | glyA | 2326871 | r | 92 |
| PurR | codA | purK | 194072 | r | 72 |
| PurR | purL | gcvP | 350625 | r | 109 |
| PurR | purB | purK | 638022 | r | 27 |
| PurR | purM | purK | 2067402 | r | 8 |
| PutA | putP | putA | 423 | r | 2 |
| PuuR | puuA | puuD | 212 | r | 1 |
| PuuR | puuR | puuA | 1003 | r | 2 |
| PuuR | puuP | puuC | 3556 | r | 3 |
| PuuR | puuP | puuB | 5045 | r | 6 |
| PuuR | puuA | puuC | 1835 | r | 3 |
| QseB | flhD | qseB | 1191629 | a | 3911 |
| QseB | flhD | qseC | 1192285 | a | 4055 |
| QseB | qseB | flhC | 1191982 | a | 3278 |
| QseB | flhC | qseC | 1192638 | a | 4226 |
| RcdA | csgE | asr | 567636 | a | 1846 |
| RcdA | asr | csgF | 568050 | a | 1914 |
| RcdA | asr | csgD | 566981 | a | 1306 |
| RcdA | asr | csgG | 568493 | a | 3683 |
| RcnR | rcnR | rcnA | 121 | r | 347 |
| RcsB | osmC | ftsZ | 1448193 | a | 180 |
| RcsB | rarA | hdeB | 2715429 | a | 3923 |
| RcsB | osmB | rarA | 402574 | a | 2955 |
| RcsB | lolA | hdeB | 2716783 | a | 2228 |
| RcsB | ftsA | gadB | 1463425 | a | 515 |
| RhaS | rhaD | rhaS | 3464 | a | 161 |
| RhaS | rhaB | rhaS | 288 | a | 159 |
| RhaS | rhaA | rhaR | 2664 | a | 66 |
| RhaS | rhaD | rhaT | 5219 | a | 14 |
| RhaS | rhaD | rhaR | 4374 | a | 11 |
| Rob | mltF | marB | 1075592 | a | 656 |
| Rob | inaA | mltF | 346329 | a | 2772 |
| Rob | nfsB | acrA | 119151 | a | 492 |
| Rob | mltF | acrA | 2208980 | a | 2528 |
| Rob | acrB | nfsB | 120367 | a | 351 |
| RstA | narH | asr | 385035 | a | 971 |
| RstA | narG | asr | 386570 | a | 717 |
| RstA | csgE | ompF | 115170 | r | 1171 |
| RstA | narJ | asr | 384328 | a | 1219 |
| RstA | csgF | ompF | 114729 | r | 1250 |
| RutR | nemA | fepB | 1100950 | r | 1956 |
| RutR | gadW | gmr | 2317147 | r | 93 |
| RutR | gmr | gadX | 2318243 | r | 119 |
| RutR | nemA | gadX | 1937229 | r | 1642 |
| RutR | gadW | nemA | 1936133 | r | 2233 |
| SdiA | gadW | ftsQ | 3557928 | a | 3750 |
| SoxS | ribA | fldB | 1700693 | a | 38 |
| SoxS | fpr | poxB | 3201477 | a | 226 |
| SoxS | inaA | fldB | 690383 | a | 586 |
| SoxS | ribA | inaA | 1009660 | a | 2635 |
| SoxS | nfsA | ptsG | 265963 | a | 1048 |
| TrpR | aroM | mtr | 2895266 | r | 139 |
| TrpR | aroM | aroH | 1379130 | r | 645 |
| TrpR | mtr | aroH | 1515090 | r | 32 |
| TrpR | mtr | aroL | 2896442 | r | 15 |
| TrpR | aroH | aroL | 1380306 | r | 2 |
| TyrR | aroM | aroF | 2330773 | r | 15 |
| TyrR | aroP | aroG | 663305 | r | 156 |
| TyrR | aroP | aroF | 2616551 | r | 24 |
| TyrR | aroM | aroP | 285101 | r | 1226 |
| TyrR | aroP | aroL | 284078 | r | 33 |
| YjiE | cysH | metL | 1241524 | a | 1213 |
| YjiE | metB | cydA | 3354446 | a | 2113 |
| YjiE | cydA | metI | 549060 | a | 3289 |
| YjiE | cysH | metN | 2662955 | a | 2610 |
| YjiE | metI | cydB | 550644 | a | 4143 |
